# Supplementary material for: Total water level driving processes influence the potential for coastal change along United States coastlines
Source: Camb Prism Coast Futur. 2025 Dec 15;3:e28. doi: 10.1017/cft.2025.10018 (PMC12780816; doi:10.1017/cft.2025.10018)
Supplement: Quadrado and Serafin supplementary material 2 — Quadrado and Serafin supplementary material [file S2754720525100188sup002.pdf]

Supporting Information to "Total Water Level Driving Processes Influence the Potential for Coastal Change along United States Coastlines"

Text S1. To separate the still water level (SWL) into relative sea level, astronomical tides, seasonality, storm surge, and sea level anomalies, we first estimate seasonality (intra-annual signal) by averaging the de-meaned and de-trended SWL observations by month and fitting a regression model that combines annual and semi-annual harmonics to these monthly SWL climatological averages. We subtract the seasonality component from the de-meaned and de-trended SWL record and implement a 15-day moving average to isolate processes occurring at time scales longer than 15 days, capturing the long-term relative sea level and sea level anomalies. To separate both processes, we apply a least-squares quadratic fit to the 15-day filtered signal to estimate the relative sea level, which is advantageous compared to a linear fit as it captures potential non-linear variations in the long-term sea level trend (Li *et al.* 2021; Wahl *et al.* 2013). Then, we subtract the long-term relative sea level from the 15-day averaged signal, and the remaining difference corresponds to sea level anomalies associated with interannual to interdecadal variability driven by changes in weather and climate patterns, current positions, and coastal-trapped waves (Dangendorf *et al.* 2021). With relative sea level, seasonality, and sea level anomalies removed from the SWL record, the remaining signal consists of astronomical tides and storm surge, a high-frequency component related to wind setup and atmospheric pressure anomalies. To separate storm surge from astronomical tides, we apply a spectral filtering method, which removes tidal bands and other low-frequency processes and replaces them with amplitude and phase estimates consistent with the nontidal continuum (Serafin & Ruggiero, 2014). Astronomical tides are retrieved from NOAA predictions from each station, including 37 harmonic constituents, and the annual/semiannual signal are removed. Finally, we reconstruct the SWL time series by adding the relative sea level, astronomical tides, seasonality, sea level anomalies, and storm surge. Using spectral filtering and only 37 harmonic constituents may cause the loss of measured SWL frequencies associated with storm surge-tide interactions that cannot be physically recreated. To retain all magnitude information from the original record, thus, a residual is calculated as the difference between the measured and reconstructed SWL time series and added back to the reconstructed SWL.

Text S2. The U.S. Atlantic and Gulf coasts database provides mean beach slope measurements, defined as the gradient between the dune toe and the shoreline based on the Mean High Water (MHW) of the NAVD88 datum (Doran et al., 2020). Following the same definition, we calculate the mean beach slope along the U.S. Pacific coast by computing the derivative from the XYZ coordinates (i.e., easting, northing, NAVD88 vertical datum) of the shoreline and dune toe positions. We exclude from the analysis all beach slopes greater than or equal to 0.12, as the Stockdon et al. (2006) empirical model was parameterized using beach slopes with average values generally smaller than this value.

Text S3. To evaluate the robustness of using static beach morphology, we conducted two case studies, one at Duck, NC, on the Atlantic coast, and another at Pensacola, FL, on the Gulf coast, as both locations have a fairly high amount of LiDAR surveys. In these case studies, we replicate our relative contribution analysis using 11 and 10 USGS LiDAR surveys, respectively at Duck and Pensacola, to test how TWL contributions change when beach slopes and dune thresholds (i.e., toe and crest elevations) are treated as static versus time-varying. Specifically, we use:

1. <u>Long-term averaged slopes and morphological thresholds:</u> estimated long-term average beach slopes and morphological thresholds at individual transects across multiple surveys and assessed TWL contributions based on these long-term mean values.

2. <u>Fully time-varying beach morphology:</u> recalculated wave runup and TWL contributions using updated beach slopes and morphological thresholds whenever survey data were available, averaging results across all “time windows” to assess average TWL relative composition during impact regimes from 1980 through 2021. For example, at Duck, NC, we used a survey from 26 November 2005 to assess the TWL relative composition during impact regimes from this date until 26 March 2008, when the next survey is available.

These case studies indicate that although TWL relative composition varies over time due to temporal variations in beach morphology, the overall average relative contributions to storm impact regimes remain consistent. Figures S2 and S3 indicate that the relative contributions from the static beach morphology approach (single-survey, main text) generally lie within the variability of the contributions associated with the multiple survey approaches (case studies,

supporting information), demonstrating that our average results using the relative contributions associated with static beach morphology are sufficient to characterize the dominant drivers of potential coastal impacts at the national scale.

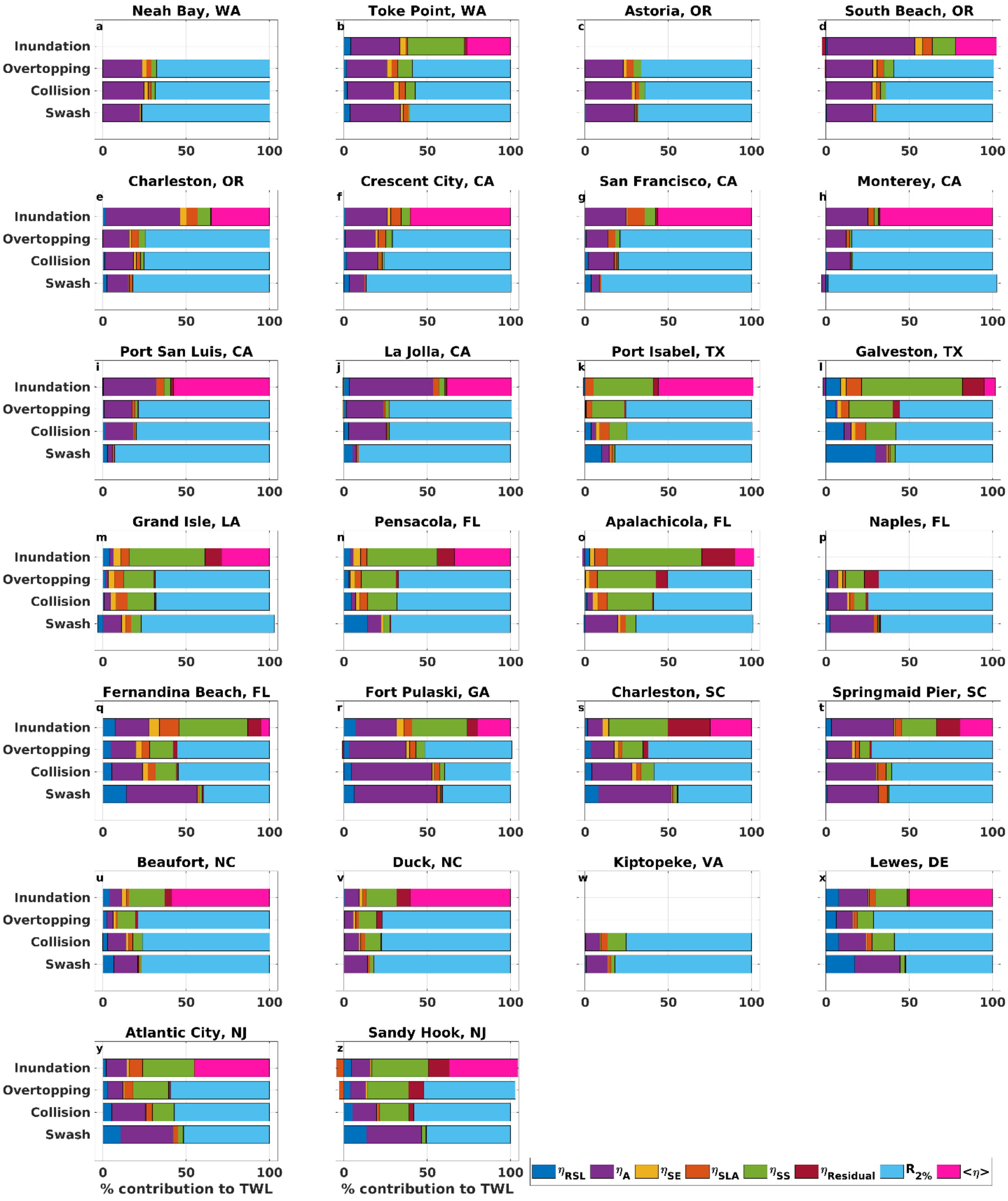

Figure S1. Average percent contribution of individual water level components to total water levels (TWLs) during swash, collision, overtopping, and inundation storm impact regimes from 1980 through 2021 at stations along the U.S. Pacific (a – j), Gulf (k – p), and Atlantic (q – z)

coastlines. SWLs are represented by the relative sea level (η_{MSL}) in dark blue, astronomical tides (η_A) in purple, seasonality (η_{SE}) in yellow, sea level anomalies (η_{SLA}) in orange, storm surge (η_{SS}) in green, and residual ($\eta_{residual}$) in red. The light blue represents wave runup ($R_{2\%}$), and the pink represents wave setup (<η>). Regimes with no events occurring are left blank.

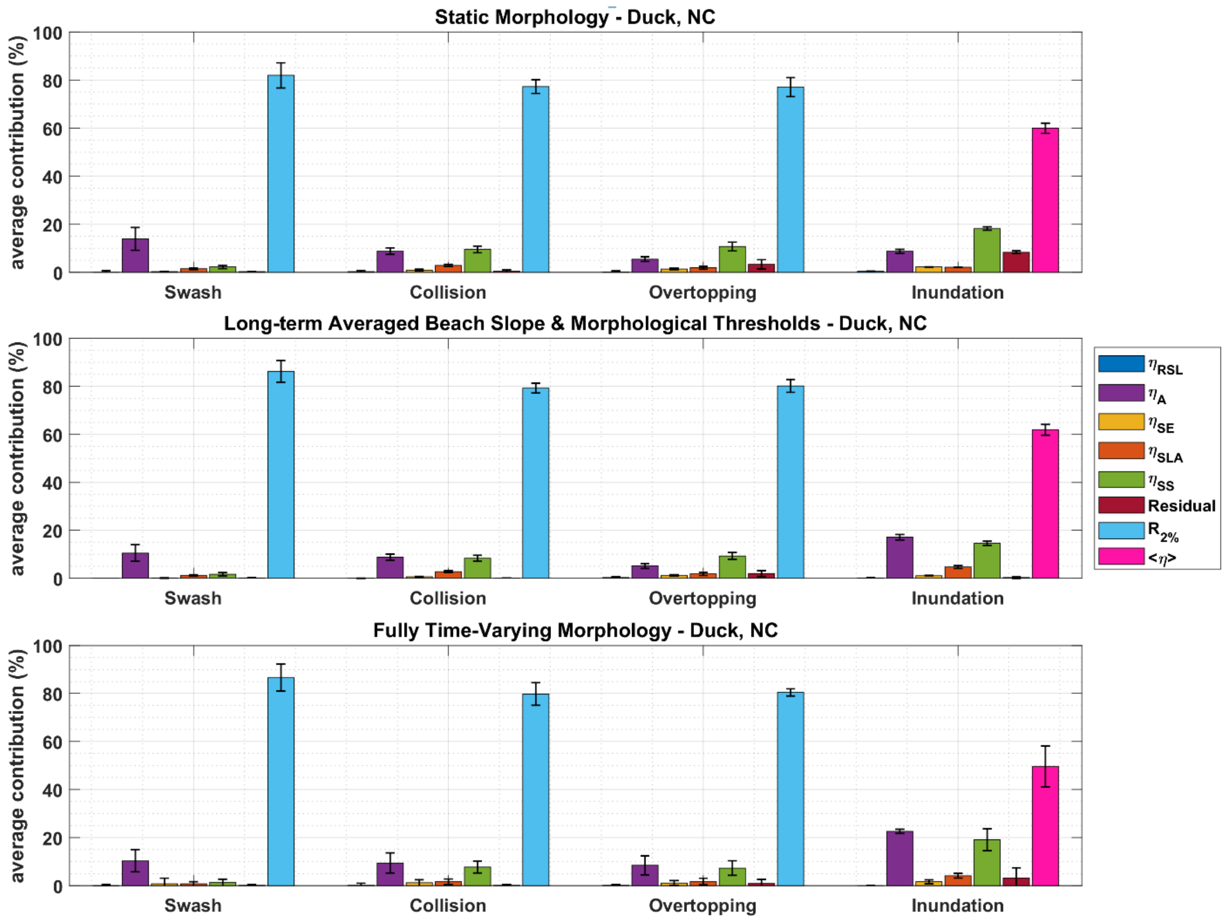

Figure S2. Comparison of average percent contribution of individual water level components to TWLs during storm impact regimes at Duck, NC, using a constant, single LiDAR survey (top panel), long-term averaged beach slopes and morphological thresholds from multiple surveys (middle panel), and fully time-varying beach slopes and morphological thresholds from multiple surveys (bottom panel). SWLs are represented by the relative sea level (η_{RSL}) in dark blue, astronomical tides (η_A) in purple, seasonality (η_{SE}) in yellow, sea level anomalies (η_{SLA}) in orange, storm surge (η_{SS}) in green, and residual ($\eta_{residual}$) in red. The light blue represents wave runup ($R_{2\%}$), and the pink represents wave setup (<η>). Error bars show ±1 standard deviation across profiles.

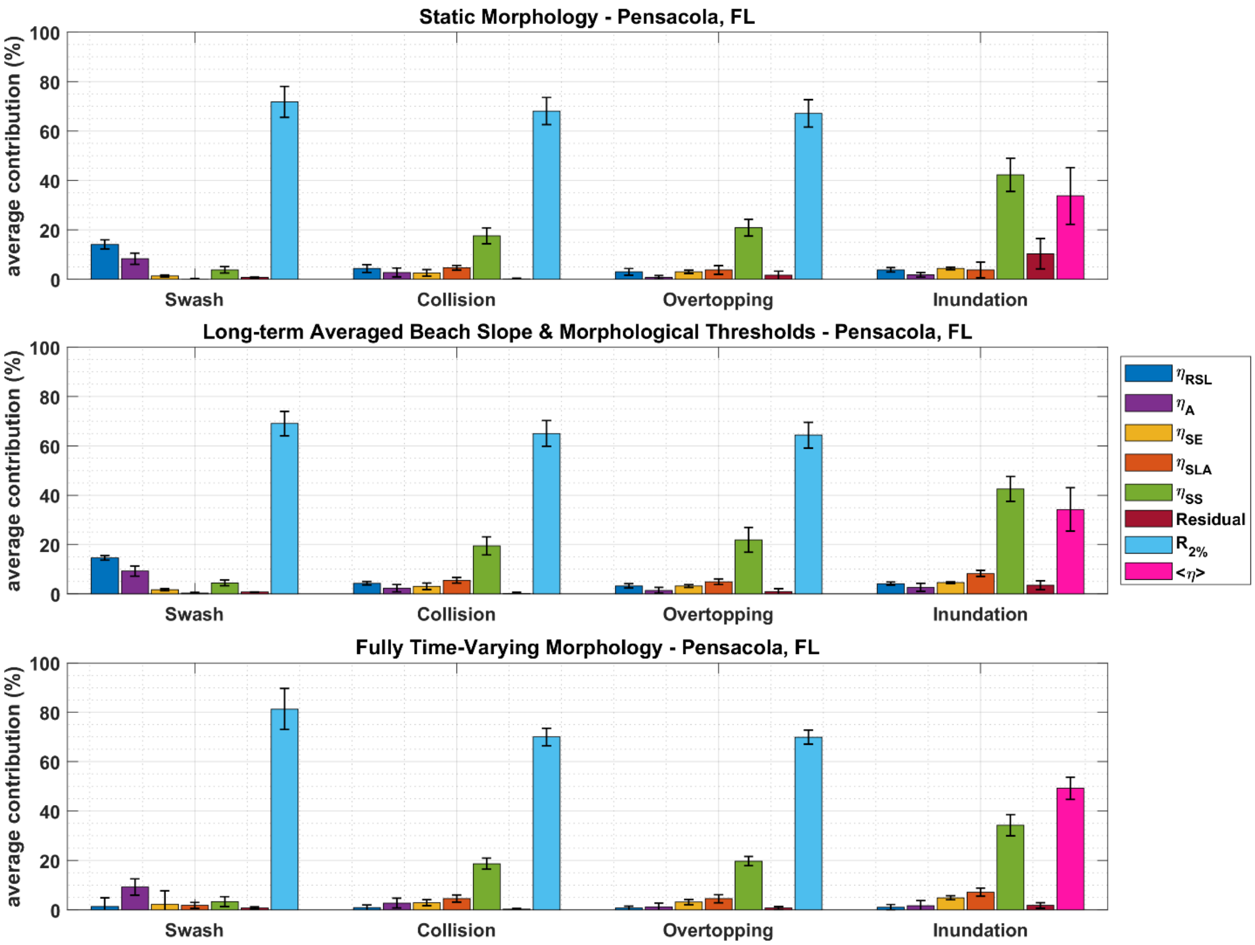

Figure S3. Comparison of average percent contribution of individual water level components to TWLs during storm impact regimes at Pensacola, FL, using a constant, single LiDAR survey (top panel), long-term averaged beach slopes and morphological thresholds from multiple surveys (middle panel), and fully time-varying beach slopes and morphological thresholds from multiple surveys (bottom panel). SWLs are represented by the relative sea level (η_{RSL}) in dark blue, astronomical tides (η_{A}) in purple, seasonality (η_{SE}) in yellow, sea level anomalies (η_{SLA}) in orange, storm surge (η_{SS}) in green, and residual ($\eta_{residual}$) in red. The light blue represents wave runup ($R_{2\%}$), and the pink represents wave setup ($\langle\eta\rangle$). Error bars show ±1 standard deviation across profiles.

Table S1. Location of coastal stretches used to obtain beach morphology data for each station. Coordinates are shown for the start and end points of each 30 km stretch. Beach morphology data were retrieved from the LiDAR-derived beach morphology surveys from the U.S. Geological Survey (USGS) (Doran *et al.* 2020; Shope *et al.* 2021). This data included alongshore-varying dune toe, dune crest, and beach slopes from beach profiles spaced 100-m apart. The fourth column lists the reference for the single Pacific coast survey or the file name, for Gulf and Atlantic coast surveys from Doran et al. (2020). The fifth column displays the year in which the morphology surveys were conducted.

Name	Start Point (latitude and longitude)	End Point (latitude and longitude)	File Name/ Reference	Survey Year
Neah Bay, WA	48.2955861, -124.6710250 | 48.2491954, -124.6997438 | 48.1813681, -124.7088087*	48.3428803, -124.6765032 | 48.2766519, -124.6808778 | 48.2246960, -124.6933773*	Shope et al. (2021)	2016
Toke Point, WA	46.572679, -124.065256	46.904694, -124.134187	Shope et al. (2021)	2016
Astoria, OR	45.976410, -123.938197	46.225573, -124.012402	Shope et al. (2021)	2016
South Beach, OR	44.422161, -124.078858	44.673803, -124.064681	Shope et al. (2021)	2016
Charleston, OR	43.357166, -124.339444	43.616280, -124.217848	Shope et al. (2021)	2016
Crescent City, CA	41.745487, -124.200424 | 41.696201, -124.142527*	41.929630, -124.204850 | 41.744557, -124.179383*	Shope et al. (2021)	2016
San Francisco, CA	37.892131, -122.635670 | 37.595890, -122.507226*	37.907309, -122.681382 | 37.778268, -122.514238*	Shope et al. (2021)	2016
Monterey, CA	36.601513, -121.889606	36.852211, -121.811760	Shope et al. (2021)	2016
Port San Luis, CA	34.907745, -120.668942	35.147218, -120.650585	Shope et al. (2021)	2016
La Jolla, CA	32.852591, -117.260873	33.116753, -117.326077	Shope et al. (2021)	2016
Port Isabel, TX	25.936619, -97.145701	26.189937, -97.176638	2018-307-CNT	2018
Galveston, TX	29.248937, -94.856221	29.458087, -94.617564	09CNT06	2009
Grand Isle, LA	29.086115, -90.223494	29.262493, -89.949476	10CNT17	2010
Pensacola, FL	29.587300, -85.045000	29.704057, -84.760211	10CNT06	2010
Apalachicola, FL	30.317168, -87.255663	30.362574, -86.947747	10CNT06	2010

Naples, FL	25.997269, -81.755949	26.304024, -81.831889	15CNT02	2015
Sandy Hook, NJ	40.209966, -74.003814	40.474461, -74.000269	17CNT01	2017
Atlantic City, NJ	39.220055, -74.637016	39.395446, -74.376528	17CNT01	2017
Lewes, DE	38.518400, -75.052700	38.803900, -75.092300	10CNT12	2010
Kiptopeke, VA	36.650100, -75.900000	36.930800, -76.016300	10CNT09	2010
Duck, NC	36.050030, -75.678110	36.319993, -75.807415	2018-308-DD	2018
Beaufort, NC	34.647700, -77.084200	34.632900, -76.528500	10CNT07	2010
Springmaid Pier, SC	33.531400, -79.031100	33.750000, -78.802900	05CNT10	2005
Charleston, SC	32.654900, -79.937300	32.810000, -79.722700	05CNT10	2005
Fort Pulaski, GA	31.949000, -80.907100	32.218800, -80.668500	05CNT10	2005
Fernandina Beach, FL	30.514500, -81.435800	30.700070, -81.426500	2016-368-DD	2016

*Multiple segments were selected to account for an approximately 30km-long coastline stretch comprising exclusively open-coast sandy beaches.

Table S2. Morphological thresholds elevations in meters defining storm impact regimes relative to NAVD88. Presented values of dune toe, dune crest, and beach slope include the average ± standard deviation at each location in this study. The relative sea level trend, with reference period, and mean tidal range, extracted from NOAA Tides and Currents Database, are also included.

ID	Location	MHHW relative to NAVD88 (m)	Dune Toe (m)	Dune Crest (m)	Average ± Std. Deviation Beach Slope	Relative Sea Level Trend (mm/year)	Time Period of Reported Relative Sea Level Trend	Tidal Range (m)
1	Neah Bay, WA	2.17	4.6 ± 0.7	8.0 ± 1.5	0.03 ± 0.02	-1.70	1934 - 2024	1.7
2	Toke Point, WA	2.482	5.0 ± 0.8	13.3 ± 2.6	0.04 ± 0.01	0.48	1973 – 2024	2.1
3	Astoria, OR	2.697	4.6 ± 1.1	15.0 ± 6.4	0.04 ± 0.02	-0.14	1925 - 2024	2.1
4	South Beach, OR	2.286	5.2 ± 0.9	10.1 ± 1.3	0.07 ± 0.02	1.79	1967 - 2024	1.9
5	Charleston, OR	2.153	3.8 ± 1.5	9.5 ± 4.8	0.07 ± 0.02	1.13	1970 - 2024	1.7
6	Crescent City, CA	1.97	4.3 ± 1.8	11.6 ± 7.3	0.06 ± 0.02	-0.77	1933 - 2024	1.5
7	San Francisco, CA	1.758	3.2 ± 0.9	17.9 ± 8.6	0.08 ± 0.02	1.98	1897 - 2024	1.2
8	Monterey, CA	1.64	3.8 ± 1.2	10.2 ± 4.3	0.06 ± 0.02	1.72	1973 - 2024	1.1
9	Port San Luis, CA	1.56	4.0 ± 1.2	14.4 ± 8.5	0.07 ± 0.02	0.98	1945 - 2024	1.1
10	La Jolla, CA	1.541	4.4 ± 1.1	12.6 ± 8.3	0.05 ± 0.02	2.02	1924 - 2024	1.1
11	Port Isabel, TX	0.223	1.8 ± 0.3	4.6 ± 1.6	0.05 ± 0.03	4.37	1944 - 2024	0.4
12	Galveston, TX	0.321	1.6 ± 0.5	3.2 ± 1.4	0.02 ± 0.02	6.65	1904 - 2024	0.3
13	Grand Isle, LA	0.215	2.4 ± 0.7	2.5 ± 1.3	0.03 ± 0.01	9.17	1947 - 2024	0.3
14	Pensacola, FL	0.27	2.1 ± 0.6	3.1 ± 0.9	0.06 ± 0.03	2.72	1923 -2024	0.4
15	Apalachicola, FL	0.285	1.7 ± 0.5	2.9 ± 1.0	0.05 ± 0.02	3.08	1967 - 2024	0.3
16	Naples, FL	0.177	1.2 ± 0.2	2.0 ± 0.5	0.06 ± 0.02	3.35	1965 - 2023	0.6
17	Fernandina Beach, FL	0.836	3.1 ± 0.6	4.8 ± 0.9	0.05 ± 0.01	2.31	1897 - 2024	1.8
18	Fort Pulaski, GA	1.054	1.7 ± 0.4	2.7 ± 1.0	0.04 ± 0.01	3.66	1935 - 2024	2.1
19	Charleston, SC	0.799	2.3 ± 0.5	3.3 ± 1.1	0.04 ± 0.02	3.51	1901 - 2024	1.6
20	Springmaid Pier, SC	0.745	1.8 ± 0.4	3.3 ± 0.6	0.08 ± 0.02	3.36	1957 - 2024	1.5
21	Beaufort, NC	0.453	2.3 ± 0.6	5.0 ± 1.5	0.06 ± 0.02	3.62	1953 - 2024	0.9
22	Duck, NC	0.457	3.3 ± 0.5	6.6 ± 1.0	0.07 ± 0.03	4.99	1978 - 2024	1.0
23	Kiptopeke, VA	0.333	2.5 ± 0.5	5.3 ± 1.9	0.05 ± 0.02	4.03	1951 - 2024	0.8
24	Lewes, DE	0.581	2.5 ± 0.5	5.0 ± 2.2	0.04 ± 0.02	3.77	1919 - 2024	1.2
25	Atlantic City, NJ	0.933	2.3 ± 0.3	4.2 ± 0.8	0.02 ± 0.01	4.25	1911 - 2024	1.2
26	Sandy Hook, NJ	0.735	3.0 ± 0.5	5.3 ± 1.5	0.04 ± 0.02	4.28	1932 - 2024	1.4

Table S3. Average relative contribution ± standard deviation of total water level (TWL) driving processes to storm impact regimes (swash, collision, overtopping, inundation) at Duck, NC, and Pensacola, FL. Results are presented for two cases (i) long-term averaged beach slope and morphological thresholds, and (ii) fully time-varying beach slopes and morphological thresholds as described in Text S3.

Location	Case	Regime	TWL Driving Process	Average Relative Contribution ± Standard Deviation (%)
Duck, NC	Long-term Average Beach Slope & Morphological Thresholds	Swash	Relative Sea Level	-0.04 ± 0.02
Duck, NC	Long-term Average Beach Slope & Morphological Thresholds	Swash	Astronomical Tides	10.57 ± 3.46
Duck, NC	Long-term Average Beach Slope & Morphological Thresholds	Swash	Seasonality	0.13 ± 0.09
Duck, NC	Long-term Average Beach Slope & Morphological Thresholds	Swash	Sea Level Anomalies	1.22 ± 0.29
Duck, NC	Long-term Average Beach Slope & Morphological Thresholds	Swash	Storm Surge	1.68 ± 0.71
Duck, NC	Long-term Average Beach Slope & Morphological Thresholds	Swash	Residual	0.22 ± 0.03
Duck, NC	Long-term Average Beach Slope & Morphological Thresholds	Swash	Wave Runup	86.21 ± 4.53
Duck, NC	Long-term Average Beach Slope & Morphological Thresholds	Collision	Relative Sea Level	0.07 ± 0.05
Duck, NC	Long-term Average Beach Slope & Morphological Thresholds	Collision	Astronomical Tides	8.82 ± 1.32
Duck, NC	Long-term Average Beach Slope & Morphological Thresholds	Collision	Seasonality	0.65 ± 0.14
Duck, NC	Long-term Average Beach Slope & Morphological Thresholds	Collision	Sea Level Anomalies	2.80 ± 0.40
Duck, NC	Long-term Average Beach Slope & Morphological Thresholds	Collision	Storm Surge	8.37 ± 1.23
Duck, NC	Long-term Average Beach Slope & Morphological Thresholds	Collision	Residual	0.07 ± 0.10
Duck, NC	Long-term Average Beach Slope & Morphological Thresholds	Collision	Wave Runup	79.23 ± 2.02
Duck, NC	Long-term Average Beach Slope & Morphological Thresholds	Overtopping	Relative Sea Level	0.30 ± 0.41
Duck, NC	Long-term Average Beach Slope & Morphological Thresholds	Overtopping	Astronomical Tides	5.16 ± 1.02
Duck, NC	Long-term Average Beach Slope & Morphological Thresholds	Overtopping	Seasonality	1.22 ± 0.31
Duck, NC	Long-term Average Beach Slope & Morphological Thresholds	Overtopping	Sea Level Anomalies	1.86 ± 0.67
Duck, NC	Long-term Average Beach Slope & Morphological Thresholds	Overtopping	Storm Surge	9.33 ± 1.44
Duck, NC	Long-term Average Beach Slope & Morphological Thresholds	Overtopping	Residual	1.98 ± 1.20
Duck, NC	Long-term Average Beach Slope & Morphological Thresholds	Overtopping	Wave Runup	80.16 ± 2.68
Duck, NC	Long-term Average Beach Slope & Morphological Thresholds	Inundation	Relative Sea Level	0.17 ± 0.21
Duck, NC	Long-term Average Beach Slope & Morphological Thresholds	Inundation	Astronomical Tides	17.12 ± 1.14
Duck, NC	Long-term Average Beach Slope & Morphological Thresholds	Inundation	Seasonality	1.17 ± 0.10
Duck, NC	Long-term Average Beach Slope & Morphological Thresholds	Inundation	Sea Level Anomalies	4.73 ± 0.60
Duck, NC	Long-term Average Beach Slope & Morphological Thresholds	Inundation	Storm Surge	14.61 ± 0.91
Duck, NC	Long-term Average Beach Slope & Morphological Thresholds	Inundation	Residual	0.33 ± 0.32
Duck, NC	Long-term Average Beach Slope & Morphological Thresholds	Inundation	Wave Setup	61.87 ± 2.23
Duck, NC	Time-Varying Beach Slope & Morphological Thresholds	Swash	Relative Sea Level	0.00 ± 0.06
Duck, NC	Time-Varying Beach Slope & Morphological Thresholds	Swash	Astronomical Tides	10.36 ± 3.24
Duck, NC	Time-Varying Beach Slope & Morphological Thresholds	Swash	Seasonality	0.77 ± 0.17
Duck, NC	Time-Varying Beach Slope & Morphological Thresholds	Swash	Sea Level Anomalies	0.75 ± 0.22
Duck, NC	Time-Varying Beach Slope & Morphological Thresholds	Swash	Storm Surge	1.33 ± 0.72
Duck, NC	Time-Varying Beach Slope & Morphological Thresholds	Swash	Residual	0.14 ± 0.07
Duck, NC	Time-Varying Beach Slope & Morphological Thresholds	Swash	Wave Runup	86.65 ± 4.17
Duck, NC	Time-Varying Beach Slope & Morphological Thresholds	Collision	Relative Sea Level	0.19 ± 0.20
Duck, NC	Time-Varying Beach Slope & Morphological Thresholds	Collision	Astronomical Tides	9.43 ± 2.55
Duck, NC	Time-Varying Beach Slope & Morphological Thresholds	Collision	Seasonality	1.20 ± 0.45
Duck, NC	Time-Varying Beach Slope & Morphological Thresholds	Collision	Sea Level Anomalies	1.59 ± 0.48
Duck, NC	Time-Varying Beach Slope & Morphological Thresholds	Collision	Storm Surge	7.71 ± 1.42
Duck, NC	Time-Varying Beach Slope & Morphological Thresholds	Collision	Residual	0.08 ± 0.31
Duck, NC	Time-Varying Beach Slope & Morphological Thresholds	Collision	Wave Runup	79.80 ± 3.43
Duck, NC	Time-Varying Beach Slope & Morphological Thresholds	Overtopping	Relative Sea Level	0.10 ± 0.15
Duck, NC	Time-Varying Beach Slope & Morphological Thresholds	Overtopping	Astronomical Tides	8.42 ± 1.67

			Seasonality	1.06 ± 0.20
			Sea Level Anomalies	1.70 ± 0.34
			Storm Surge	7.32 ± 1.38
			Residual	0.93 ± 0.46
			Wave Runup	80.46 ± 2.53
		Inundation	Relative Sea Level	-0.30 ± 0.00
			Astronomical Tides	22.60 ± 0.15
			Seasonality	1.60 ± 0.01
			Sea Level Anomalies	4.10 ± 0.03
			Storm Surge	19.13 ± 0.15
			Residual	3.24 ± 0.04
			Wave Setup	49.63 ± 0.37
Pensacola, FL	Long-term Average Beach Slope & Morphological Thresholds	Swash	Relative Sea Level	14.60 ± 0.91
			Astronomical Tides	9.23 ± 2.03
			Seasonality	1.68 ± 0.39
			Sea Level Anomalies	0.28 ± 0.36
			Storm Surge	4.49 ± 1.15
			Residual	0.73 ± 0.14
			Wave Runup	69.00 ± 4.95
		Collision	Relative Sea Level	4.35 ± 0.70
			Astronomical Tides	2.31 ± 1.47
			Seasonality	3.10 ± 1.32
			Sea Level Anomalies	5.50 ± 1.12
			Storm Surge	19.45 ± 3.67
			Residual	0.25 ± 0.42
			Wave Runup	65.03 ± 5.22
		Overtopping	Relative Sea Level	3.26 ± 0.89
			Astronomical Tides	1.46 ± 1.20
			Seasonality	3.19 ± 0.57
			Sea Level Anomalies	4.92 ± 1.16
			Storm Surge	21.92 ± 5.03
			Residual	0.94 ± 1.17
			Wave Runup	64.31 ± 5.19
		Inundation	Relative Sea Level	4.21 ± 0.60
			Astronomical Tides	2.67 ± 1.63
			Seasonality	4.55 ± 0.35
			Sea Level Anomalies	8.30 ± 1.20
			Storm Surge	42.52 ± 5.05
			Residual	3.51 ± 1.81
			Wave Setup	34.24 ± 8.77
	Time-Varying Beach Slope & Morphological Thresholds	Swash	Relative Sea Level	1.33 ± 0.28
			Astronomical Tides	9.23 ± 2.80
			Seasonality	2.22 ± 0.57
			Sea Level Anomalies	1.76 ± 0.57
			Storm Surge	3.32 ± 1.61
			Residual	0.80 ± 0.18
			Wave Runup	81.34 ± 5.66
		Collision	Relative Sea Level	0.85 ± 0.39
			Astronomical Tides	2.73 ± 1.97
			Seasonality	2.90 ± 1.27
			Sea Level Anomalies	4.57 ± 1.11
			Storm Surge	18.70 ± 3.91
			Residual	0.24 ± 0.42
			Wave Runup	70.02 ± 5.92

Overtopping	Relative Sea Level	0.73 ± 0.31
	Astronomical Tides	1.21 ± 1.35
	Seasonality	3.15 ± 0.82
	Sea Level Anomalies	4.48 ± 0.88
	Storm Surge	19.76 ± 4.56
	Residual	0.71 ± 0.72
	Wave Runup	69.96 ± 6.32
Inundation	Relative Sea Level	1.11 ± 0.28
	Astronomical Tides	1.58 ± 1.37
	Seasonality	4.89 ± 0.72
	Sea Level Anomalies	7.15 ± 1.41
	Storm Surge	34.27 ± 5.75
	Residual	1.78 ± 1.41
	Wave Setup	49.23 ± 8.58

Table S4. Results of one-way ANOVA tests evaluating differences in TWL magnitudes across storm impact regimes and across coastal regions. The ANOVA across regimes compares TWL magnitudes among swash, collision, and overtopping regimes within seven individual coastal regions. The ANOVA across regions compares TWL magnitudes across seven coastal regions within the swash, collision, and overtopping regimes. The inundation regime is excluded from the ANOVA testing because only a few hours of inundation are detected at most individual locations, resulting in a considerably smaller sample size compared to the other regimes.

ANOVA	Regions	F-statistic (ratio variance between groups to variance within groups)	p-value
Across Regimes within Regions	Pacific Northwest (PNW)	40.6	4.54×10^{-6}
	California (CA)	27.3	3.44×10^{-5}
	West Gulf (W Gulf)	10.3	1.15×10^{-2}
	East Gulf (E Gulf)	103.8	2.22×10^{-5}
	Northeast (NE)	32.1	6×10^{-4}
	Northern Southeast (N-SE)	52.4	4.42×10^{-4}
	Southern Southeast (S-SE)	41.7	2.80×10^{-5}
Across Regions within Regimes	Swash	191.4	5.98×10^{-16}
	Collision	39.3	1.03×10^{-9}
	Overtopping	9.9	6.82×10^{-5}

Table S5. Average ± standard deviation, and median magnitudes of total water level (TWL), still water level (SWL), and wave runup ($R_{2\%}$), in meters, during swash, collision, and overtopping regimes across the United States coastlines. Results are presented for seven regions, distinguished by distinct coastal geography, guided by U.S. climate regions (Karl and Koss 1984).

Region	Regime	Physical Process	Metric	Elevation (m)
Pacific Northwest (PNW)	Swash	TWL	Average ± Standard Deviation	3.0 ± 0.4
			Median	2.9
		SWL	Average ± Standard Deviation	1.6 ± 0.4
			Median	1.5
		R2%	Average ± Standard Deviation	1.4 ± 0.3
			Median	1.3
	Collision	TWL	Average ± Standard Deviation	4.9 ± 0.8
			Median	5.0
		SWL	Average ± Standard Deviation	2.3 ± 0.5
			Median	2.3
		R2%	Average ± Standard Deviation	2.6 ± 0.8
			Median	2.4
	Overtopping	TWL	Average ± Standard Deviation	5.7 ± 1.2
			Median	5.4
		SWL	Average ± Standard Deviation	2.6 ± 0.4
			Median	2.5
		R2%	Average ± Standard Deviation	3.2 ± 1.2
			Median	2.6
California (CA)	Swash	TWL	Average ± Standard Deviation	2.2 ± 0.4
			Median	2.2
		SWL	Average ± Standard Deviation	0.8 ± 0.4
			Median	0.8
		R2%	Average ± Standard Deviation	1.4 ± 0.3
			Median	1.4
	Collision	TWL	Average ± Standard Deviation	3.6 ± 0.9
			Median	3.4
		SWL	Average ± Standard Deviation	1.3 ± 0.3
			Median	1.3
		R2%	Average ± Standard Deviation	2.3 ± 0.8
			Median	2.2

	Overtopping	TWL	Average ± Standard Deviation	4.9 ± 1.0
			Median	4.9
		SWL	Average ± Standard Deviation	1.4 ± 0.3
			Median	1.4
		R2%	Average ± Standard Deviation	3.5 ± 1.0
			Median	3.5
West Gulf (W-Gulf)	Swash	TWL	Average ± Standard Deviation	0.4 ± 0.1
			Median	0.4
		SWL	Average ± Standard Deviation	-0.1 ± 0.1
			Median	-0.1
		R2%	Average ± Standard Deviation	0.5 ± 0.1
			Median	0.4
	Collision	TWL	Average ± Standard Deviation	1.7 ± 0.5
			Median	1.6
		SWL	Average ± Standard Deviation	0.3 ± 0.3
			Median	0.3
		R2%	Average ± Standard Deviation	1.3 ± 0.5
			Median	1.4
	Overtopping	TWL	Average ± Standard Deviation	2.9 ± 1.2
			Median	2.5
		SWL	Average ± Standard Deviation	0.7 ± 0.5
			Median	0.7
		R2%	Average ± Standard Deviation	2.2 ± 1.1
			Median	1.9
East Gulf (E-Gulf)	Swash	TWL	Average ± Standard Deviation	0.4 ± 0.1
			Median	0.3
		SWL	Average ± Standard Deviation	0.0 ± 0.1
			Median	0.0
		R2%	Average ± Standard Deviation	0.4 ± 0.1
			Median	0.4
	Collision	TWL	Average ± Standard Deviation	1.7 ± 0.6
			Median	1.6
		SWL	Average ± Standard Deviation	0.4 ± 0.3
			Median	0.3

		R2%	Average ± Standard Deviation	1.3 ± 0.5
			Median	1.2
	Overtopping	TWL	Average ± Standard Deviation	2.8 ± 0.7
			Median	2.7
		SWL	Average ± Standard Deviation	0.7 ± 0.3
			Median	0.7
		R2%	Average ± Standard Deviation	2.0 ± 0.6
			Median	2.0
Northeast (NE)	Swash	TWL	Average ± Standard Deviation	0.9 ± 0.1
			Median	0.9
		SWL	Average ± Standard Deviation	0.2 ± 0.1
			Median	0.2
		R2%	Average ± Standard Deviation	0.7 ± 0.1
			Median	0.7
	Collision	TWL	Average ± Standard Deviation	2.8 ± 0.5
			Median	2.8
		SWL	Average ± Standard Deviation	0.8 ± 0.3
			Median	0.9
		R2%	Average ± Standard Deviation	1.9 ± 0.5
			Median	1.9
	Overtopping	TWL	Average ± Standard Deviation	4.0 ± 0.8
			Median	4.1
		SWL	Average ± Standard Deviation	0.9 ± 0.7
			Median	1.0
		R2%	Average ± Standard Deviation	3.1 ± 0.8
			Median	2.8
North Southeast (N-SE)	Swash	TWL	Average ± Standard Deviation	1.0 ± 0.1
			Median	1.0
		SWL	Average ± Standard Deviation	0.1 ± 0.1
			Median	0.2
		R2%	Average ± Standard Deviation	0.8 ± 0.1
			Median	0.8
	Collision	TWL	Average ± Standard Deviation	2.7 ± 0.6
			Median	2.7
		SWL	Average ± Standard Deviation	0.5 ± 0.2
			Median	0.5

		R2%	Average ± Standard Deviation	2.2 ± 0.5
			Median	2.1
	Overtopping	TWL	Average ± Standard Deviation	4.7 ± 0.7
			Median	4.7
		SWL	Average ± Standard Deviation	0.8 ± 0.3
			Median	0.8
		R2%	Average ± Standard Deviation	3.9 ± 0.7
			Median	3.9
South Southeast (S-SE)	Swash	TWL	Average ± Standard Deviation	1.0 ± 0.1
			Median	1.0
		SWL	Average ± Standard Deviation	0.3 ± 0.1
			Median	0.3
		R2%	Average ± Standard Deviation	0.6 ± 0.1
			Median	0.6
	Collision	TWL	Average ± Standard Deviation	2.3 ± 0.6
			Median	2.2
		SWL	Average ± Standard Deviation	0.8 ± 0.3
			Median	0.8
		R2%	Average ± Standard Deviation	1.6 ± 0.5
			Median	1.5
	Overtopping	TWL	Average ± Standard Deviation	3.3 ± 0.8
			Median	3.2
		SWL	Average ± Standard Deviation	0.9 ± 0.4
			Median	0.9
		R2%	Average ± Standard Deviation	2.3 ± 0.8
			Median	2.3

Table S6. Average ± standard deviation percentile of morphological thresholds relative to the total water level, for swash, collision, and overtopping, and the dynamic still water level, for the inundation regime across the U.S. Pacific, Gulf, and Atlantic coastlines.

Region	SWASH	COLLISION	OVERTOPPING	INUNDATION
	MHHW relative to TWL percentile	Dune toe relative to TWL percentile	Dune crest relative to TWL percentile	Dune crest relative to dynamic SWL percentile
Pacific Coast	36.4 ± 11.3	92.0 ± 4.8	99.5 ± 0.7	99.9 ± 0.0
Gulf Coast	32.5 ± 12.9	99.2 ± 0.9	99.8 ± 0.2	99.9 ± 0.0
Atlantic Coast	53.9 ± 10.4	97.5 ± 4.2	99.2 ± 1.4	99.6 ± 0.6

References

Dangendorf S, Frederikse T, Chafik L, Klinck JM, Ezer T and Hamlington BD (2021) Data-driven reconstruction reveals large-scale ocean circulation control on coastal sea level. *Nature Climate Change 2021 11:6* 11(6), 514–520. https://doi.org/10.1038/s41558-021-01046-1.

Doran KS, Long JW, Birchler JJ, Brenner OT, Hardy MW, Morgan KLM, Stockdon HF and Torres ML (2020) *Data Release - Lidar-derived Beach Morphology (Dune Crest, Dune Toe, and Shoreline) for U.S. Sandy Coastlines*. St. Petersburg, FL: USGS. Retrieved from https://coastal.er.usgs.gov/data-release/doi-F7GF0S0Z/

Karl TR and Koss WJ (1984) Regional and national monthly, seasonal, and annual temperature weighted by area, 1895-1983. *Historical Climatology Series 3-3*.

Li S, Wahl T, Barroso A, Coats S, Dangendorf S, Piecuch C, Sun Q, Thompson P and Liu L (2021) Contributions of Different Sea-Level Processes to High-Tide Flooding Along the U.S. Coastline. *Journal of Geophysical Research: Oceans* 127(7), e2021JC018276. https://doi.org/10.1029/2021JC018276.

Quadrado GP and Serafin KA (2024) The Timing, Magnitude, and Relative Composition of Extreme Total Water Levels Vary Seasonally Along the U.S. Atlantic Coast. *Journal of*

Geophysical Research: Oceans 129(9), e2023JC020557. https://doi.org/10.1029/2023JC020557.

Serafin KA and Ruggiero P (2014) Simulating extreme total water levels using a time-dependent, extreme value approach. *Journal of Geophysical Research: Oceans* 119(9), 6305–6329. https://doi.org/10.1002/2014JC010093.

Shope JB, Erikson LH, Barnard PL and Storlazzi CD (2021) Modeled extreme total water levels along the U.S. west coast. U.S. Geological Survey. https://doi.org/10.5066/P95FBGZ1.

Wahl T, Haigh ID, Woodworth PL, Albrecht F, Dillingh D, Jensen J, Nicholls RJ, Weisse R and Wöppelmann G (2013) Observed mean sea level changes around the North Sea coastline from 1800 to present. *Earth-Science Reviews* 124, 51–67. https://doi.org/10.1016/J.EARSCIREV.2013.05.003.
