# Supplementary material for: Total water level driving processes influence the potential for coastal change along United States coastlines
Source: Camb Prism Coast Futur. 2025 Dec 15;3:e28. doi: 10.1017/cft.2025.10018 (PMC12780816; doi:10.1017/cft.2025.10018)
Supplement: Quadrado and Serafin supplementary material 1 — Quadrado and Serafin supplementary material [file S2754720525100188sup001.docx]

**RESPONSE TO REVIEWERS**

Additional Editor-in-Chief Comments to Author:

I have now received comments and recommendations from three reviewers, and have reviewed these along with the overall recommendation of the Handling Editor. All three of the reviewers are positive about the overall contribution of this paper to the subject, and I agree that this manuscript (when revised) is of interest to the readership of Coastal Futures and will be a valuable addition to the Special Issue on Coastal Extremes.

All of the reviewers recommend revisions, with two recommending minor revisions and one recommending major revisions. Given the quantity and scope of the recommended revisions, I support the Handling Editor's recommendation of major revisions, highlighting the need to reconsider the approach to beach slope and include a discussion of the limitations of the work. I acknowledge the challenges in defining a representative slope from complex beach profiles; however, I believe that this is important and is linked to the suggestion of one reviewer to consider a sensitivity analysis as part of this study. I would be interested to see a more detailed exploration of the beach slope definition, and its impact on the results, in the revised manuscript. For other recommended revisions, I encourage the authors to engage with the detailed comments provided by each reviewer.

To summarise, I believe that this paper will make a valuable contribution to knowledge after the revisions are completed. I look forward to reading the revised manuscript.

Response: We thank the Editor-In-Chief for recognizing the value and relevance of our study to the Coastal Futures readership and the Special Issue on Coastal Extremes. We have carefully considered all editorial and reviewer feedback and have strengthened the rationale for our beach slope definition choice and its implications for coastal impact occurrence. In response to the suggestions regarding sensitivity analysis and the use of static morphology, we conducted two case studies using multiple LiDAR morphology surveys to assess how a time-varying morphology approach influences our results, and we discuss it in the main text as well as in the supporting information material. These revisions are detailed in our responses to individual reviewer comments.

Handling Editor's Comments to Author:

Handling Editor: Power, Hannah

Comments to the Author:

In addition to the reviewers' comments, I would emphasise the need to reconsider your approach to beach slope when calculating wave runup as this is a critical parameter that makes a large difference to the estimations. Further, I would strongly encourage you to consider including a discussion of the limitations of your work in the main body of the manuscript rather than including it as a supplmement

Response: We thank the Handling Editor for the constructive feedback. We revisited our approach to defining beach slope given its critical importance to wave runup estimation and concluded that using the mean beach slope between the dune toe and shoreline best aligns with the physical processes driving the coastal impacts in our work, supporting our choice with previous studies in the methods section. We have also conducted two case studies using multiple LiDAR morphology surveys to assess how time-varying beach slopes could potentially influence our results, and our findings corroborate our single, static survey approach to represent beach slopes. The findings of our case studies are now included in the manuscript in the main text and in the supporting information material. As suggested, we have also moved and expanded the discussion of our study’s limitations from the Supporting Information to the main text (i.e., Section 4.3, Limitations and Assumptions), improving transparency and contextualizing the potential implications of our approach. These and other updates are described in detail throughout our responses to the reviewer’s comments below.

Reviewer(s)' Comments to Author:

Reviewer: 1
Comments to the Author
Overview
This study evaluates the spatial variability, magnitude, and relative composition of total water level at various locations along US coastlines. The authors classified the study regions into 4 regimes and quantified the probability of each regime to experience impacts. The authors investigate the importance of different components and aspects of the Sallenger regimes contained in the total water level.

The study objective and the adopted methodology are mostly clear. The authors presented their results in a clear way, carried out a well-structured discussion on their findings, and contextualized their findings with some existing research. The manuscript is well-written and well-structured.

I recommend publication after minor revision

Response: We appreciate the reviewer’s positive feedback regarding the manuscript’s structure and clarity. We thank Reviewer #1 for their comments and the attention given to this manuscript. Below, please find responses that address each concern raised.

Major comments:
1.1) This study can benefit from some additional comparisons with more recent previous studies addressing TWL and various components to flooding.

Response: We thank the reviewer for this suggestion and acknowledge the importance of connecting our results to recent hazard-focused studies. To address this, we incorporated comparisons with several studies, including Parker et al. (2023), Quadrado & Serafin (2024), and Serafin et al. (2017) from lines 347 – 350:

*“While previous studies identified waves as the main driver of extreme TWLs (i.e., annual maximum, 100-year event) along the U.S. coastlines (e.g., Parker et al. 2023; Quadrado and Serafin 2024; Serafin et al. 2017), our results show that even more frequent events, like those in the swash regime, are also wave-dominated.”*

To support our finding that high relative sea level contributions to TWLs during swash are related to rising seas in the Gulf, we cite Talke (2025) from lines 358 – 363:

*“For instance, the highest relative sea level contribution to TWLs during swash (29.5%) occurs in Galveston, TX, which has the second-highest relative sea level rise rate across stations (i.e., 6.65 mm/year; NOAA CO-OPS, 2025). This is consistent with evidence that diurnally dominated, microtidal regions like the Gulf are highly sensitive to sea level rise, as even small increases in sea levels can rapidly lengthen the periods during which water levels remain above flooding thresholds relative to tidal datums (Talke, 2025).”*

We refer to Cohn et al. (2019) from lines 374 – 378 to highlight the importance of TWL relative composition to morphology response during the collision regime:

*“Differences in the proportion waves and SWLs influence TWLs can lead to distinct coastal responses during collision. For example, field observations and simulations in the PNW show that wave-driven dune toe accretion can occur on low-sloping beaches when the dynamic SWL remains below the dune toe during collision (Cohn et al., 2019).”*

From lines 378 – 383, we cite the recent work by Leung et al. (2024), who used collision and overtopping hours as proxies for erosion and flooding hazards:

*“Variations in TWL relative composition also introduce uncertainty in coastal hazard projections. On the U.S. Pacific coast, a probabilistic hazard assessment reveals that wave runup and astronomical tides are the main contributors to collision (Leung et al. 2024). Variability in wave and SWL magnitude adds uncertainty to collision and overtopping projections that can exceed uncertainty associated with sea level rise through the 21st century, since small TWL changes around regime thresholds can determine impact occurrence (Leung et al., 2024).”*

We also included this study in the discussion from lines 429 – 432:

*“On the Pacific coast, beaches near the California-Oregon border and Northern Washington are more susceptible to collision and overtopping than adjacent regions due to their steep beach slopes (> 0.1) and relatively low backshore feature toes (Leung et al., 2024).”*

We also cite the work by Hsu et al. (2023), which evaluates TWL relative contributions during tropical cyclone events based on numerical simulations, including an assessment of extreme TWL composition and duration associated with the exceedance of collision and overtopping thresholds. Specifically, on lines 423 – 429, we discuss why the Atlantic and Pacific coastlines are more likely to experience impacts than the Gulf across our study sites by discussing the following:

*“**This reflects more energetic wave climates (Ahn et al., 2020; Lehmann et al., 2017) and higher mean tidal ranges (Table S2) along the Atlantic and Pacific, which increase the probability of threshold exceedance.* *For instance, TWL simulations during Hurricanes Matthew and Dorian show that more locations along the U.S. Atlantic coast experienced dune toe and crest exceedances when peak TWLs were considered, rather than the combination of peak storm surge and wave runup alone, demonstrating the importance of tides to impact occurrence (Hsu et al., 2023).”*

1.2) The study is conducted regionally and I can understand why. However, some discussion on variability within regions (not captured) from the present approach is needed. For example beach slope, dune variability etc. Also am I correct that no station were used on the east of Florida south of Fernandina?  Why?

Response: We appreciate the reviewer for raising this point. We acknowledge that grouping by broader regions (e.g., California) may mask subregional differences (e.g., North CA vs. South CA), but our regional scale was chosen to emphasize large-scale patterns across the U.S. coastlines, while the presentation of station-level TWL composition results still capture subregional and site-specific variability (e.g., Figure 4). Additionally, by incorporating alongshore variability in beach morphology across 30km-long coastal segments, our analysis accounts for local variability in morphology and reduces potential risks of masking important local patterns. We highlight local patterns that differ from the regional averaging in some paragraphs in the results and discussion sections:

- Lines 293-297: “*The top 3 largest contributors to TWLs in the swash regime are wave runup (70%), astronomical tides (20%), and relative sea level (6%), although at some sites contributions from storm surge and/or sea level anomalies exceed those from relative sea* *level, like Charleston, Grand Isle, Springmaid Pier, and Duck (e.g., Figure 4b, f, j-k).”*
- Lines 312 – 315: “*At several sites, storm surge contributions during inundation exceed regional averages by up to 24%, for example, at Toke Point and South Beach in the Pacific, Galveston and Apalachicola on the Gulf, and from Charleston to Fernandina Beach on the Atlantic (Figure S1).”*
- Lines 411-414: *“Storm surge contribution peaks during the inundation regime, but at some locations, this reflects only a few storm events, like Hurricanes Isaac (2012) and Ida (2021) at Grand Isle, LA, where storm surge exceeded wave setup contribution (Figure 4).”*

It is correct that we do not include any water level stations on the east coast south of Fernandina Beach, FL. This is because these stations do not meet our selection criteria for record length (≥ 30 years) and completeness (≥ 80% of hourly data over the available record). For example, 8720218 Mayport (Bar Pilots Dock), 8721604 Trident Pier, and 8723214 Virginia Key hourly records start in 1995 (27 years), 1994 (28 years), and 2010 (11 years), respectively. At each station, we further evaluate hourly completeness each year, and exclude years with < 80% hourly data, which further reduces the record length at these locations.

1.3) The study could benefit from a sensitivity analysis.
1.3a) Example: Only a single lidar survey could be used that would not represent the mean or possibly even typical conditions of a dynamic area.

Response: We thank the reviewer for raising this point. While our analysis includes the spatial variability in beach slope for our defined coastal stretches to reduce uncertainties from assuming spatially averaged morphology, we recognize that wave runup is highly sensitive to temporal changes in beach slope as well and our approach provides a snapshot of morphology in time. The use of a single, static survey could also affect the occurrence of impact regimes which are tied to the elevation of morphological thresholds.

To evaluate the robustness of using static beach morphology, we conducted two case studies, one at Duck, NC, on the Atlantic coast, and another at Pensacola, FL, on the Gulf coast, as both locations have a fairly high amount of LiDAR surveys. In these case studies, we replicate our relative contribution analysis using 11 and 10 USGS LiDAR surveys, respectively at Duck and Pensacola, to test how TWL contributions change when beach slopes and dune thresholds (i.e., toe and crest elevations) are treated as static versus time-varying. Specifically, we use:

(i) **Long-term averaged slopes and morphological thresholds:** estimated long-term average beach slopes and morphological thresholds at individual transects across multiple surveys and assessed TWL contributions based on these long-term mean values – results displayed below in Figure 1 for Duck, NC, and Figure 2 for Pensacola, FL.
(ii) **Fully time-varying beach morphology:** recalculated wave runup and TWL contributions using updated beach slopes and morphological thresholds whenever survey data were available, averaging results across all “time windows” to assess average TWL relative composition during impact regimes from 1980 through 2021. For example, at Duck, NC, we used a survey from 26 November 2005 to assess the TWL relative composition during impact regimes from this date until 26 March 2008, when the next survey is available – results displayed below in Figure 1 for Duck, NC, and Figure 2 for Pensacola, FL.

This analysis shows that although the relative composition of TWLs varies over time due to temporal variations in beach morphology, the overall average relative contributions to storm impact regimes remain consistent. Figures 1 and 2 indicate that the relative contributions from the static beach morphology approach (single-survey) generally lie within the variability of the contributions associated with the multiple survey approaches (case studies). These findings, thus, demonstrate that our average results using the relative contributions associated with the most recent static beach morphology that wasn’t a post-storm condition are sufficient to characterize the dominant drivers of potential coastal impacts and support the robustness of our approach at the national scale, especially considering that not all sites have morphological surveys available at the same temporal resolution as Duck, NC, and Pensacola, FL.

Additionally, our case study conclusions are consistent with **Kim et al. (2023),** who found that the Stockdon et al. (2006) wave runup model performed just as well when using a constant, pre-storm beach slope compared to using time-varying slopes throughout a storm at Duck, NC, with the latter providing little improvement. This further demonstrates that the broader-scale patterns we find here are consistent and provide robust estimates of TWL contributions, despite temporal variations in beach slope.

We refer to this case study now in the methods section, from lines 219 to 229, to support the robustness of our approach. We also discuss it in the limitations and assumptions section (lines 442 – 450), now added to the discussion in the main manuscript (Section 4.3), and in the Supporting Information material, with a description of the case study in Text S3 and results displayed in Figures S2 and S3.

**Figure 1.** Comparison of average percent contribution of individual TWL water level components to TWLs during storm impact regimes at Duck, NC, using a constant, single LiDAR survey (top panel), long-term averaged beach slopes and morphological thresholds from multiple surveys (middle panel), and fully time-varying beach slopes and morphological thresholds from multiple surveys (bottom panel). SWLs are represented by the relative sea level (η_RSL_) in dark blue, astronomical tides (η_A_) in purple, seasonality (η_SE_) in yellow, sea level anomalies (η_SLA_) in orange, storm surge (η_SS_) in green, and residual (η_residual_) in red. The light blue represents wave runup (R_2%_), and the pink represents wave setup (<η>). Error bars show ±1 standard deviation across profiles.

**Figure 2.** Comparison of average percent contribution of individual TWL water level components to TWLs during storm impact regimes at Pensacola, FL, using a constant, single LiDAR survey (top panel), long-term averaged beach slopes and morphological thresholds from multiple surveys (middle panel), and fully time-varying beach slopes and morphological thresholds from multiple surveys (bottom panel). SWLs are represented by the relative sea level (η_RSL_) in dark blue, astronomical tides (η_A_) in purple, seasonality (η_SE_) in yellow, sea level anomalies (η_SLA_) in orange, storm surge (η_SS_) in green, and residual (η_residual_) in red. The light blue represents wave runup (R_2%_), and the pink represents wave setup (<η>). Error bars show ±1 standard deviation across profiles.

1.3b) Example: the beach slope used in the Stockdon formula seems problematic. The authors use the gradient from the dune toe to the MHW. That slope will certainly be different than the true foreshore slope. The back beach is often “flat” or may even have a negative gradient if there is a pronounced berm. Why not choose a definition more commensurate with the foreshore slope… like +/- 1 m either side of mean water, or +/- 2 m.

Response: We understand the reviewer’s concern regarding the beach slope definition, as wave runup is commonly parameterized using foreshore beach slope. However, we chose to use the mean beach slope because the foreshore slope can vary daily due to wave action, and storm waves often flatten the foreshore profile, altering its slope (Palmsten & Holman, 2012; Stockdon et al., 2012). In addition, extreme water levels typically act over the whole beach face, rather than just the foreshore. Since we are assessing storm impacts across the whole beach system, not only the swash zone, the mean beach slope definition provides a more representative metric of the evaluated processes.

Palmsten & Holman (2012) examined the predictive skill of the Stockdon et al. (2006) runup model during dune erosion events using four different definitions of beach slope: 1) a time-varying mean beach slope (gradient between the SWL line and the base of the dune), 2) a time-constant, pre-storm mean beach slope (also from the SWL line and the dune toe), and 3-4) two time-varying foreshore beach slopes (MWL ± 1 std. deviation and MWL ± 2 std. deviation). They found that wave runup predictions for R_16_ (i.e., 16% exceedance level) on the time-independent mean beach slope were slightly better than those based on the foreshore slope. This supports the conclusion of Stockdon et al. (2007) that the initial mean beach slope is a reasonable definition for beach slope in the absence of time-varying slopes. Palmsten & Holman (2012) also emphasize that the mean beach slope metric is easier to estimate than the MWL ± 1 or 2 std. deviation slopes, which is important given the national scale of our study and the need for consistent reproducibility across locations.

We strengthened our justification for our beach slope choice in the methods section, including more details about how we treat beach slope values that are outside the range of those used to parameterize the Stockdon et al. (2006) formulation (lines 174 – 184):

*“To calculate wave runup, we use the mean beach slope, defined as the gradient from the dune toe to the shoreline (approximated by the Mean High Water (MHW) of the NAVD88 datum; Text S2). We use this metric over the foreshore beach slope, as the latter varies daily with wave conditions, extreme water levels typically act over the whole beach face, and storm waves often flatten the foreshore slope (Palmsten and Holman 2012; Stockdon et al. 2012). Moreover, an experimental study showed that pre-storm mean beach slopes yielded more accurate runup predictions during dune erosion events than foreshore slopes, while also being easier to estimate consistently at large spatial scales (Palmsten and Holman 2012). Because the Stockdon et al. (2006) empirical runup model was parameterized using beach slopes with average values generally < 0.12, we exclude all beach profiles with beach slopes exceeding this upper limit to remain within the model’s applicability range.”*

1.3c) Example; Do all beaches used have dunes? La Jolla is mostly backed by cliffs for example. What is done in these scenarios and does the Stockdon formula make sense with how the slope is being defined?

Response: We thank the reviewer for this question. Coastal segments were manually selected to ensure they represent open coast sandy beaches, and we did not include any segments backed by cliffs or riprap, except for La Jolla, CA. Along La Jolla’s coastal segment, there are short sections backed by a localized seawall and ripraps, representing a total of 2.7km out of the 30km long coastal segment, which is ~9% of the coastal segment. While we did not check each profile point by point when selecting sites at the national scale, our broader screening aimed to include only sandy beaches. With this comment, we noted that La Jolla includes short stretches of engineered features. This coastal segment also includes more locations than just La Jolla, so it is not backed by cliffs (Figure 3). The methods used by Doran et al. (2020) and Shope et al. (2021) specify that where dunes are absent, berms are used, with the berm crest and toe representing dune crest and toe, respectively. Shope et al. (2021) also states that where engineered backshore features are present, the structure top is used as the dune crest, and the dune toe is identified either a few meters seaward or at the nearest beach profile inflection point (for seawalls) or at the junction where sand meets the base of the structure (for ripraps) (Shope et al., 2021). These datasets, however, do not provide metadata identifying which features were used to derive each toe and crest estimate, which is a limitation of the datasets used.

Figure 3a below shows the full extent of the coastal segment manually selected for La Jolla, CA, and the locations of the riprap (red areas) and the seawall (yellow area). For some of these segments, the Shope et al. (2021) dataset does not provide data points, further reducing the proportion of coastline that is backed up by engineered structures. We apply the Stockdon et al. (2006) runup model only within its range of applicability by excluding profiles with beach slopes > 0.11, as highlighted in response to comment 3.2 for Reviewer 3. Given that the engineered sections at La Jolla comprise a small fraction of the total segment and that we use a slope cut off, using Stockdon remains a reasonable approach in our study.

**A**

**B**

**Figure 3.** Map of the La Jolla, CA, coastal segment selected for the analysis (approximately 30 km long) (a). Blue pins mark the start and end coordinates of the coastal segment. Red and yellow areas indicate the locations of ripraps and a localized seawall, respectively. Example of dune toe elevations extracted from cross-shore profiles along the La Jolla segment from the Shope et al. (2021) dataset for the largest riprap area highlighted in panel a (b).

1.4) The writing throughout is passive.

Response: We understand the concern regarding passive writing. We have carefully reviewed the manuscript with this suggestion in mind and made selective edits to increase the use of the active voice where it improved clarity. We also note that in some cases, we still use the passive voice to maintain conciseness and stay within the word count limit of the journal.

Minor comments:

1.5) Line 86: “Gulf of Mexico” ◊ “Gulf of America” now.  Also fix Figure 1

Response: We have updated Figure 1 to include both terms, “Gulf of America” and “Gulf of Mexico”, ensuring clarity for all readers while maintaining the internationally recognized nomenclature given Coastal Futures’ international readership.

1.6) Line 136: TWL and SWL were defined before, use the abbreviations instead.

Response: We revised the sentence on line 123 and now use only the acronyms.

1.7) Line 138:  This equation is so basic that it is not needed

Response: We recognize that the equation is simple, but we prefer to retain it because it explicitly links the variables for readers who may not be familiar with the TWL definition.

1.8) Line 155: The Stockdon formula is used for runup but it is far from “perfect”.  Some studies have shown the R2% from this approach underestimate erosion (Palmsten and Holman 2012) AND this type of simple formula lacks explicit treatment of the dune and foreshore geometry (Park and Cox, 2016) beyond the foreshore slope.

Response: We agree with the reviewer that the Stockdon et al. (2006) empirical runup formulation has limitations. As noted by Palmsten and Holman (2012), the conventional R_2%_ statistic is not always the most skillful predictor of dune erosion, with their laboratory experiments showing that dune erosion correlates more strongly with a less extreme wave runup exceedance level (i.e., R_16_). However, this finding reflects the sensitivity of dune erosion to the choice of exceedance statistic rather than a systematic underestimation by the Stockdon model itself, and R_16_ has not been parameterized or validated consistently at large spatial scales. In our study, the objective is not to predict dune erosion directly, but to use runup exceedance as a proxy for when impacts may occur, making the use of R_2%_ suitable in this context.

Park and Cox (2016) also highlighted limitations of the Stockdon formulation. They confirmed that the model performs well when wave runup is confined to the foreshore (when foreshore slope is used), but noted that it may underpredict (overpredict) wave runup when the foreshore slope (dune slope) is used and wave runup is acting on the dune system. Their work introduces a new wave runup parameterization and modifications to the Stockdon formulation (i.e., alternative slope definitions and a berm-width reduction factor), but these were developed with synthetic data from idealized cross-shore profiles, calibrated and verified using the same dataset, and rely on detailed morphological metrics not consistently available at national scales.

Despite these known limitations, we chose to use Stockdon et al. (2006) R_2%_ formulation for several reasons:

1) The empirical formulation was developed using observational datasets from various sandy beaches, including locations at or close to our study sites, like Duck, NC, and Scripps Beach in La Jolla, CA, and Agate Beach, OR, making it effective across different morphodynamic states and wave conditions – this information is given in the manuscript from lines 152 to 155.
2) The Stockdon et al. (2006) predictive skill has been tested multiple times, both with overwash proxies that incorporate beach topography observations (e.g., Matias et al., 2012; Plant and Stockdon, 2012; Stockdon et al., 2007) or using direct measurements of runup (e.g., Gomes da Silva et al., 2020; Guza and Feddersen, 2012; Kim et al., 2023; Stockdon et al., 2014, 2007), with most studies concluding that this empirical model is skillful.
3) Our study is a nation-scale assessment. While more complex R2% formulations (e.g., incorporating berm width, dune geometry, or alternative exceedance levels) may improve local-scale predictions, they rely on data that are not available consistently at all our sites and are beyond the scope of this work.

For these reasons, we believe that the Stockdon et al. (2006) formulation is the most appropriate choice to address our goals. It provides a simple, empirically validated, and widely adopted approach that balances accuracy with feasibility at national scales. We acknowledge that other wave runup formulations, such as those available in the Python Wave Runup Toolbox (Leaman, 2020), are similarly straightforward and easy to implement, and may perform better under specific wave conditions, since existing formulations perform best within the specific range of conditions for which they were parameterized (Itzkin et al., 2025). However, uncertainties across alternative empirical wave runup formulations may be comparable to those of Stockdon et al. (2006). A comprehensive assessment of these uncertainties will be an important focus of our future work.

1.9) Line 202: “To identify whether an hourly TWL is in a specific regime”. This phrase is not clear.

Response: We revised this sentence (now in line 200) to *“**To classify hourly TWLs into storm impact regimes,, …”* for improved clarity.

1.10) Figure 2: the legend and the text are not easily readable in subplots (1) and (5).

Response: We enlarged the legends and text in subplots 1 and 5 in Figure 2 to improve readability.

1.11) Lines 249, 251: define n. I supposed the number of ANOVA tests (7 then 3). Or degrees of freedom?

Response: We thank the reviewer for pointing out the missing clarification of “n” in the text. We have now clarified that the numbers refer to the levels being compared in each ANOVA. Specifically, in the first case, there are three levels (the storm impact regimes within a region), and in the second case, there are seven levels (the regions within a regime). The revised sentences now read (lines 246 – 250):

*“First, to evaluate whether average TWL magnitudes differ across regimes within a region, we perform seven ANOVAs, one for each region, using the regionally averaged TWL magnitudes for each regime (number of levels = 3). Second, to assess whether average TWL magnitudes differ across regions within a regime, we perform three ANOVAs, one for each regime, using the regional TWL averages (number of levels = 7).”*

1.12) Figure 3: The y-scale is different between subplots. Suggesting unifying the y-scale for each variable for easier comparison.

Response: We updated Figure 3 following this suggestion. Specifically, we standardized the x- and y-axes across the collision and overtopping regime subplots (Figures 3b, 3c, 3e, 3f, 3h, and 3i). For the swash regime, we also standardized axes across its subplots (Figures 3a, 3d, and 3g) but kept them distinct from the other regimes, since swash presents much smaller TWL, SWL, and R_2%_ magnitudes and higher probability densities compared to the collision and overtopping regimes.

1.13) Figure 4: The legend is too small. The caption is too long.

Response: We updated the legend on Figure 4 to improve readability and shortened the caption to make it more concise.

1.14) Line 359: I imagine this statement comes from the particular locations where data were extracted. However, blanket statements on slopes being “steeper” for the broad regions is misleading.

Response: We agree that there is an important distinction between findings from our study sites and broader regional patterns. To address this concern, we revised this sentence, now on lines 352 - 353, to: *“…where beach slopes are among the steepest across our sites (Table S2)…”.*

Reviewer: 2

Comments to the Author
The manuscript by Quadrado & Serafin examines the importance of considering not only total water level (TWL) magnitude but also its composition when determining coastal impacts using Sallenger’s storm impact scale. The authors emphasise that understanding spatial variations in TWL composition is important for prioritising which factors to consider in preparing for coastal hazards and, consequently, for improving coastal adaptation planning. Furthermore, they compare TWL magnitudes within each impact regime across multiple locations, assessing the probability of each site experiencing each regime.
I find the article to be of high quality, well structured, clearly written, and supported by good and valuable results presented in detail. The authors acknowledge the limitations of their approach in a clear manner. The study yields interesting findings regarding TWL composition and local variability, of relevance to both the coastal research community and coastal managers. Overall, my impression is very positive, and I recommend it for publication. Nonetheless, I do have some suggestions that may help to improve the manuscript.

Response: We are pleased the reviewer found the manuscript to be a positive contribution. We thank the reviewer for their recommendations to improve the clarity of our manuscript. Below, we address each of the specific comments individually.  

Specific comments:
2.1) Line 102: You use the term overtopping instead of overwash. Most studies retain Sallenger's original term. Is there a reason to use overtopping instead of overwash? While I don't see a problem with your choice, please clarify in the text that this is your terminology change and that Sallenger originally used overwash.

Response: While Sallenger (2000) originally used the term “overwash”, we opted to use “overtopping” to describe this regime because it is consistent with more recent studies (e.g., Almar et al., 2021; Gallien, 2016; Hames et al., 2023; Leung et al., 2024; Oliver et al., 2024) and conceptually aligns with Sallenger’s definition, as it describes water flowing over the dune or berm crest, leading to dune erosion and potential sediment deposition landward. To avoid confusion, we now clarify in the text that “overtopping” refers to the regime originally termed “overwash” by Sallenger (2000) (line 195).

2.2) Line 143: Not all NOAA station data are complete (minimum 75% hourly data over the record). Please specify how missing data were handled. Were they interpolated? Please clarify this in the text to improve methodological transparency.

Response: We thank the reviewer for bringing this to our attention. The correct percentage is 80%, not 75%, and we have now clarified this in the manuscript (line 130). Data completeness is assessed at two levels. First, stations are only included if they have at least 80% hourly completeness across the ~40 years we evaluate. Second, within each selected station, we exclude any individual year with less than 80% hourly completeness. We chose this threshold because 20% of a given year corresponds to approximately 73 days, and missing data over > 2 months could bias results towards the periods with available data, which may mean we’d miss impacts. For years that meet the 80% criterion, gaps remain unfilled and are simply excluded from the analysis. We revised the text to ensure transparency (lines 129 – 133):

*“Stations are selected based on record length (*≥*30 years), geographic location (*≥*50 km apart), and completeness (*≥*80% of hourly data between 1980 and 2021). At each station, we further evaluate hourly completeness each year, and exclude years with < 80% hourly data, since missing 20% or more (i.e.,* ≈ *73 days) can bias findings toward times of the year with available data and potentially exclude extreme events causing impacts.”*

2.3) Line 198: Consider whether the same term "flooding" should apply to both overtopping/overwash and inundation. For the first, overtopping may be more precise. If both are described as flooding, the reader might be confused. Changing the term flooding to inundation for the second could be a possibility (although I'm not sure if it is the correct term in this case). But then a definition distinguishing flooding from inundation would be needed.

Response: We thank the reviewer for raising this concern. To align with the definitions proposed in Sallenger (2000) and Stockdon et al. (2007), we have characterized the overtopping regime as a proxy for dune erosion with landward sediment transport. We further clarified that overtopping is associated with flood potential as during this regime, water levels intermittently exceed the dune crest, while during inundation, dunes are continuously submerged, and thus serve as a true proxy for flooding. We also maintained “overtopping” as the regime name as explained in response 2.1 and retained inundation as the proxy for flooding. The revised sentence (lines 194-199):

*“Collision serves as a proxy for dune toe erosion, while overtopping (originally named overwash in Sallenger (2000)), is a proxy for* *dune erosion with landward sediment transport with flood potential, as water levels intermittently overtop the dune. The most severe regime is inundation, measured using the dynamic SWL (i.e., SWL + wave setup) instead of TWL, which serves as a flooding proxy since the beach and the dunes are continuously submerged.”*

2.4) Figure 2: Looking at the methodology of figure 2, would it be possible to include an analysis of the duration each regime dominates annually? I'm curious about it. Especially how many hours per year the inundation regime occurs, and whether this duration changes over time for each location.

Response: We appreciate the suggestion and agree that understanding the variability of annual impact hours for each regime is valuable. We decided not to include this analysis in the present manuscript, as we have prepared a separate study in which we assess linear trends in impact hours associated with storm impact regimes and conduct a sensitivity analysis to quantify the relative influence of TWL drivers and beach slope on impact hour variability. This second manuscript is currently under review in *Coastal Engineering*, and is available as a preprint:

*Quadrado, G., & Serafin, K. Rising Impact Hours on Sandy Beaches Linked to Total Water Level Variability Along U.S. Coastlines. SSRN.* [*http://dx.doi.org/10.2139/ssrn.5394558*](http://dx.doi.org/10.2139/ssrn.5394558)

In Figures 2 through 5 of this preprint, we present the number of hours per year that each regime occurs across the same 26 study sites considered in the present manuscript.

2.5) Figure 3: There seem to be negative SWL values, why are these not included in the plots? Or are these data being excluded? Also, since axis ranges are very similar (e.g., 8 and 10), using the same axis limits across panels (same x for all, same y for each column) would greatly improve comparability.

Response: We appreciate the reviewer’s thoughtful question on this point. Negative values do occur in the SWL empirical distributions, although infrequently during the threshold exceedances which is what we display, as negative values could be tied to the specific datum of presentation or reflect a combination of negative sea level anomalies, storm surge, and tidal values. We have updated the axes in all subplots of Figure 3 to include these negative values and to improve comparability across variables and regimes. Please refer to response 1.12 for a more detailed explanation of the updates in Figure 3.

2.6) Line 279: Please clarify whether values refer to PNW or CA, or if they are a median across both. If the latter, I suggest presenting median TWL values for each region separately. For the Gulf regions, this distinction seems less critical given their similarity.

Response: Following the reviewer’s suggestion, we now distinguish between the PNW and CA regions and clarify that both Gulf regions are included when describing magnitude changes from swash to collision and from collision to overtopping (lines 269 – 274):

*“The PNW and CA exhibit the highest TWLs and standard deviations across regions, with averages increasing by 1.9 m and 0.8 from swash to collision and collision to overtopping in the PNW, and by 1.4 m and 1.3 m in CA for the same transitions. In contrast, the Gulf regions show the lowest TWLs and narrowest distributions but still experience absolute increases comparable to those along the Pacific coast, with average TWLs increasing by 1.3 m from swash to collision and 1.2 m from collision to overtopping.”*

2.7) Line 299: Please clarify what you mean by "tides." Does this refer exclusively to astronomical tides? If so, by looking at the figure (although I understand the statistics represent all the stations), it seems surprising to have more RSL than SS (since you mention 4% for RSL, I assume SS is less than this). Please clarify this in text.

Response: We now specify in line 294 that we are referring to “astronomical tides”. During the swash regime, average contributions from RSL to the TWL can be larger than those from storm surge. Across all stations, storm surge contributes 2% during swash, which is smaller than the contribution from RSL (~6%). Sea level anomalies also contribute, on average, 2% to TWLs during swash. At the local level, however, storm surge and sea level anomalies contributions can exceed those of the national average. We have revised the text to clarify the largest contributors to TWLs during swash and highlight the occurrence of local variability (lines 293 - 297):

*“The top 3 largest contributors to TWLs in the swash regime are wave runup (70%), astronomical tides (20%), and relative sea level (6%), although at some sites contributions from storm surge and/or sea level anomalies exceed those from relative sea level, like Charleston, Grand Isle, Springmaid Pier, and Duck (e.g., Figure 4b, f, j-k).”*

2.8) Line 300: Could you specify in the text which exceptions you refer to in Figure 4b–c? They are not visually apparent to me.

Response: For this result, we are only referring to the swash regime, and we have updated this sentence to add specific examples of the process contributions that may be different in the examples given from the broad statement as suggested, from lines 293 - 297, in the same sentence referenced in response 2.7 above.

2.9) Figure 4: What do the values below 0 and above 100 mean in the plots? Values below 0 presumably represent negative contributions, but how does the greatest relative sea level trend at Grand Isle produce a negative contribution to TWL? Similarly, clarify the meaning of values above 100. These points are currently unclear.

Response: The values below 0 represent negative contributions, as individual SWL processes (i.e., relative sea level, seasonality, sea level anomalies, tides, storm surge) can lower the TWL. When such negative contributions occur, other processes contribute positively and compensate for the negative contribution so that the sum of individual TWL contributors always equals 100%, making processes seem to contribute over 100% in Figure 4 of our manuscript.

It is true that the negative contribution of RSL to the swash regime at Grand Isle, LA, may seem counterintuitive, since this site has the highest relative sea level rise trend across the CONUS. The negative contribution arises from the way the long-term trend is fitted, with the “zero relative sea level” centered near the middle of the record, so values in the early part of the series fall below this reference. When all events are averaged, these early negative values can yield an average negative contribution even though the long-term relative sea level trend is positive. The negative contributions are, thus, a consequence of the method used to estimate long-term relative sea level and to calculate contributions to the TWL, which excludes the datum from the calculations.

**Figure 4.** Water level record from NOAA water level station 8761724 Grand Isle, LA with long-term relative sea level (η_RSL_) from 1980 to 2021. The η_RSL_ is shown relative to the Mean Lower Low Water (MLLW, blue; top panel), North American Vertical Datum of 1988 (NAVD88, orange; middle panel), and without a reference datum (yellow; bottom panel). The red dotted horizontal line indicates η_RSL_ = 0 in all panels.

2.10) Figure 5: I really like this figure. In line with my earlier comment on Figure 2, could you also present regime occurrence in hours/year? For example, on average, how many hours per year does each location experience inundation? While you mention something in line 371, a dedicated figure showing temporal evolution would be more informative and could also help visualise the increasing RSL impact (as discussed from line 374 onward).

Response: Please, refer to response 2.4 for a more detailed comment about this suggestion.

Minor corrections:
2.11) Line 78: The statement on the lack of real-time measurements linking coastal change with hourly TWLs is somewhat inconsistent with your own approach, where you combine different datasets (wave hindcast, measured water levels, LiDAR) to do exactly that. Using separate datasets is not a problem in itself, especially since you address this in the limitations section. The issue may be the use of the term “datasets.” Consider rephrasing to “separate models (or simulations) for water levels and beach morphology” to clarify the intended meaning.

Response: We would like to clarify that by “real-time measurements” we were referring to direct observations of coastal change in response to TWLs. For example, from video cameras that capture TWLs impacting the beach during a storm. By “separate datasets” we meant indirect approaches to observe coastal change, which rely on combining different data sources as we do in our work and as done in other studies that use modeling approaches. We have revised the introduction to better contextualize our work, and the specific sentence mentioned in this comment has been removed.

2.12) Methods: The water level datum is not specified, though I assume it is NAVD88 (same as for beach slope). Please confirm and state this explicitly. Also, the length of each dataset is not stated. Only on line 206 you defined the temporal scale for TWL. This information should be given earlier in the text.

Response: We now specify our study period in the introduction (line 110), and the datum is specified in the methods section (line 126).

2.13) Line 205: Add letters in the text and on each panel of figure 2 to clearly reference locations in the figure, or refer to the numbering in figure 2 when describing locations in the text (line 205 and subsequent sentences).

Response: We agree that including letters or numbers in the text helps guide the reader through Figure 2 and our workflow, and we have incorporated this suggestion in the Methods section (lines 200 – 212).

2.14) Line 332: Consider rewriting as: “Figure 5 displays the empirical percentiles of TWL (for swash, collision, and overtopping regimes) or dynamic SWL (for the inundation regime) corresponding to…” Also, please review and adjust the figure 5 legend accordingly for precision.
Response: We have revised this sentence as suggested (lines 327 – 329):

*“Figure 5 shows empirical percentiles of TWL (for swash, collision, and overtopping regimes) or dynamic SWL (for the inundation regime) at site-specific morphological thresholds, estimating the likelihood of those magnitudes.”*

2.15) Finally, I would strongly encourage you to make your code freely and readily available, and not only “upon request”.

Response: We appreciate the reviewer’s encouragement to make our code and data freely available. To enhance transparency, we now include an example of our workflow for Duck, NC, available in a publicly available data repository on GitHub (<https://github.com/gabrielleQuadrado/TWLComposition-during-ImpactRegimes>). A DOI-linked, frozen version of this repository will be created and released upon publication through Zenodo. The main input file used in the shared script is not yet publicly available in this repository, as we are awaiting confirmation from IHCantabria on whether the Global Ocean Waves (GOW) datasets they provided can be released. Regarding the data, the underlying observational and model datasets (i.e., NOAA water level stations, GOW2.0 wave hindcast, USGS LiDAR) are publicly available from their respective sources. Additional processed data shown in the figures could be shared upon request.

Reviewer: 3

Comments to the Author
Summary:
This manuscript quantifies differences in the drivers of total water levels (TWLs) across US sandy beaches. Wave hindcast and water level data from 26 locations are used to calculate hourly TWL time series. TWLs are compared to LiDAR-extracted morphology to determine the amount of time spent in Sallenger Storm Impact Regimes.
TWL composition varies spatially, temporally, and across Sallenger Regimes. Wave runup is found to be the main driver of TWLs in swash, collision, and overtopping regimes across all regions. In overtopping and (rare) inundation regimes, storm surge and tidal contribution grows – with storm surge playing a larger role in the Atlantic and Gulf Coasts, compared to Tides in the Pacific. Atlantic and Pacific coasts experience more frequent coastal impacts than the Gulf Coast due to their more energetic wave climates, despite higher morphological thresholds in the Pacific.  The Gulf Coast experiences rarer but more severe storm surge contributions due to hurricane events.  
Overall evaluation:
This manuscript addresses a valuable contribution to coastal hazard literature. Similar analyses have been conducted on a regional-scale but this is the first assessment that is conducted for the entire continental US.
The paper is well-written, methods and results are clearly presented. I find this paper makes a meaningful contribution to the literature, but could be strengthened, particularly in the discussion. I outline these points in the comments below.

Recommendation:
This paper makes a valuable contribution to coastal hazards literature by expanding analysis of the relative contributions of TWL components in driving coastal hazards across the continental US. I recommend major revisions (1) to clarify the methodology/ assumptions surrounding TWL calculation for the different morphologies and (2) strengthening the discussion by expanding analysis of non-stationarity in the observed record and implications for future impacts, as well as connecting back to existing literature. I encourage resubmission once these issues are addressed.

Response: We thank the reviewer for their positive assessment of our manuscript. We appreciate the recognition that our work makes to the coastal hazard literature. We value their suggestions for strengthening the discussion and have carefully considered their recommendations. Each specific comment is addressed below.
Major comments:
3.1) Line 176-186: Please reference Table S3 in this section to help the reader understand the different morphologic setting of each site. If there is room, the authors could put this information in the main paper rather than hidden in the supplemental as it directly informs the TWL calculations and storm impact regime results.

Response: We now refer to both Tables S1 and S2 in line 169 when mentioning local beach morphology characteristics. We appreciate the suggestion to move this into the main text, but given word count and display item limits, we opted to keep these details in the supporting information material. However, we note that the average elevations of the morphological thresholds are also presented in Figure 5, although we do not refer to this figure in this line, as it is within the Methods section and Figure 5 shows our results.

3.2) Related to the comment above, the Stockdon run up formula is designed for gently sloping to intermediate beaches (β< 0.12) backed by sandy dunes. How does this analysis deal with transects with steeper beach slopes? Did you also ensure all the relevant transects are actually backed by dunes rather than cliffs, riprap, infrastructure, other beach types that would not be appropriate to use the Stockdon formula on?

Response: We thank the reviewer for raising this concern. We only include beach profiles with beach slopes β < 0.12, as this is approximately the upper limit of the average beach slopes used to parameterize the Stockdon et al. (2006) equation. This is now specified in lines (181 - 184): *“Because the Stockdon et al. (2006) empirical runup model was parameterized using beach slopes with average values generally < 0.12, we exclude all beach profiles with beach slopes exceeding this upper limit to remain within the model’s applicability range.”* Please refer to responses 1.3b, 1.3c and 1.8 for detailed discussions on backing beach morphology and the appropriateness of the Stockdon et al. (2006) empirical model.

3.3) Line 371: It would be interesting to have a deeper exploration of the non-stationarity within the observed record. How are figures 4 & 5 different if you break up the record in different time periods? Alternatively, could authors create a figure that highlights comparisons with the past that you allude to (e.g., show that 67% of the swash hours occur between 2000-2022 for Grand Isle. How does this value compare to other locations?).

Response: We appreciate the suggestion and agree that understanding the temporal variability of contributions and impact hours for each regime per year is valuable. We do not include analyses of temporal variability of impact hours across regimes in the present study because we have prepared a separate manuscript in which we assess linear trends in impact hours associated with storm impact regimes, which is currently under review and available as a preprint. Please refer to response 2.4 for more details about the second manuscript, which you can also access from the reference below:

*Quadrado, G., & Serafin, K. Rising Impact Hours on Sandy Beaches Linked to Total Water Level Variability Along U.S. Coastlines. SSRN.* [*http://dx.doi.org/10.2139/ssrn.5394558*](http://dx.doi.org/10.2139/ssrn.5394558)

Moreover, because updated, time-varying morphological datasets are not consistently available across all our sites, we do not evaluate time-varying TWL contributions (Figure 4) or TWL or dynamic SWL percentiles of morphological thresholds representative of impact regimes (Figure 5). Thus, our analysis focuses on long-term averages of TWL composition and impact probability. In response 1.3a, we further discuss that results based on our static morphology approach are consistent with those based on time-varying beach morphology in case study locations with a fairly high availability of LiDAR surveys, supporting our decision to focus solely on long-term patterns in this manuscript.

3.4) Line 376: Authors state that results indicate that coastal hazards will increase with SLR. Can they be more explicit about what their analysis of TWL contributions suggests for future impacts across different regions and regimes? How will the relative contributions of TWLs change in different regions & regimes in the future due to SLR? Are there trends in the observed data?

Response: We thank the reviewer for this thoughtful question. Our analysis focuses on historical TWL contributions to impact regimes, a novel analysis at the national scale, and while we do not explicitly project future changes, our findings provide valuable insights into how sea level rise may influence future impacts across regions and regimes. From our results, we can infer that the contribution from relative sea level will likely increase with rising seas, especially during swash, which has the lowest threshold for TWL exceedance, and is therefore the first regime to show increases in impact hours. This finding is formally discussed in our preprint reference (please see response 2.4 for further details), in which we focus on long-term trends in swash, collision, overtopping, and inundation impact hours. In Quadrado and Serafin, 2025, preprint, we show that sandy beaches along the Gulf and Atlantic coasts exhibited increasing swash and collision impact hours from 1980 through 2021, with the strongest trends associated with the swash regime. This suggests that, as sea levels continue to rise, impact regimes will be gradually affected, making locations more likely to experience coastal impacts. We also discuss potential causes to such increases in impact hours in our preprint, beyond sea level rise, that are relevant to the question raised here.

To address this concern in the present manuscript, we now discuss the potential impact of rising seas to coastal impacts frequency based on our results and existing literature (lines 363 – 366): *“Collectively, these results suggest that rising seas could increase the frequency of coastal impacts by raising baseline water levels, which increases threshold exceedance likelihood (Almar et al. 2021; Hague et al. 2020; Hames et al. 2023; Sweet and Park 2014; Taherkhani et al. 2020)”.*

We also acknowledge the importance of smaller TWL contributors to coastal impacts in the paragraph from lines 384 to 398, where we discuss how sea level anomalies (i.e., interannual and interdecadal) and seasonality (i.e., intraannual variability) may influence the timing and frequency of impacts, providing additional insights into the temporal scales that modulate coastal hazard occurrence.

3.5) Discussion: I think the manuscript could benefit from having a paragraph referring back to the previous literature that explored relative contributions of TWLs in the US / globally that they described in the introduction (lines 61-77, lines 111-120). Is this work in line with previous studies? How does this study expand from previous studies?

Response: Previous studies of TWL in the U.S. and globally have primarily focused on extreme events or specific return periods (e.g., 100-year event), while our work evaluates TWL relative composition in the context of storm impact regimes. While not directly comparable to these studies, our findings are broadly consistent with earlier studies in showing that waves tend to dominate in contribution to TWLs along open sandy coasts (e.g., Melet et al., 2018; Parker et al., 2023; Quadrado & Serafin, 2024 Serafin et al., 2017; Vitousek et al., 2017).

We recognize that adding a paragraph referring back to these previous studies could strengthen the discussion. Instead of adding a separate paragraph, we have incorporated some of these references where appropriate to acknowledge prior work, while keeping the discussion concise, focused on the patterns associated with impact regimes, and avoiding redundancy with the introduction. For example, from lines 347 to 350 we added: “*While previous studies identified waves as the main driver of extreme TWLs (i.e., annual maximum, 100-year event) along the U.S. coastlines (e.g., Parker et al. 2023; Quadrado and Serafin 2024; Serafin et al. 2017), our results show that even more frequent events, like those in the swash regime, are also wave-dominated.”* Also, you can refer to response 1.1 for a more detailed description of the comparison of our findings to other recent studies, including some of the studies we mention in the introduction, such as Cohn et al. (2019), Hsu et al. (2023), and Theuerkauf et al. (2014).

This revision more clearly situates our findings within existing work while highlighting how our study expands on it, reinforcing the link between our results and previous findings described in the introduction (e.g., lines 86-107).

Minor comments:
3.6) Line 89-93 : “…models are typically applied at specific locations and times as they become computationally expensive for spatial scales of thousands of kilometers... integrate detailed model insights across broader spatial [and temporal] scales” Add a description for timescales that are generally computationally expensive.

Response: We have revised this sentence to better describe the time scale for which these models are generally computationally expensive (lines 63 – 65): *“…process-based models are usually applied to specific events at specific locations as they become computationally expensive at hourly resolutions over spatial domains of hundreds of kilometers and temporal scales from months to decades.”*

3.7) Line 96: Leung et al., 2024 (https://doi.org/10.1029/2024EF005523) is another recent example of a hazard proxy analysis, which may be relevant for your discussion of impacts in the PNW.

Response: We thank the reviewer for the suggestion, and we have incorporated this reference in the introduction and discussion sections of our manuscript (i.e., line 72; lines 379 - 383; lines 429 – 432).

3.8) Figure 2: in box 6 it appears some text on your x axis was cut off.
Figure 2: in box 6- you describe the colors in your figure caption, but why not match the colors in box 5 and box 6, and the visual legend in box 5 to maintain consistency?
Figure 2: box 4 has two contrasting titles describing the ‘average magnitude of TWL’  versus the ‘average wave runup’ – you describe this in the caption, but – Why? Why not just show the TWL?
Figure 2 : You could also add the dune toe and dune crest elevations to the plot in box 4, similar to in box 3.

Response: We have incorporated most of the suggestions into Figure 2. Below we provide justifications for the suggestions we did not incorporate:

- We have changed the numbers to letters to identify our workflow steps, so that box 1 now becomes box A, and so on.
- For boxes E and F, we have added a common legend to both figures in box F.
- In box D, we chose not to show the average TWL magnitude because our goal is to highlight that in this step of our workflow, we calculate an average magnitude for each individual TWL driving process by year at each individual beach profile. We illustrate this step by using wave runup magnitudes during the collision regime as an example. We added a black dotted line to this plot that indicates the average wave runup magnitude across beach profiles to address the reviewer’s concern.
- In box D, we chose not to add dune toe and dune crest elevations because we are representing average wave runup magnitudes at multiple beach profiles at once, in contrast to box 3, where we show the TWL magnitude for a single beach profile as an example.

3.9) Line 234 - : should write your methods / analysis in past tense: “we performed… we extracted… and computed… Sites were then grouped… ect.”

Response: We appreciate the reviewer’s suggestion. We chose to retain the present tense in the Methods section as a stylistic preference, which is acceptable given that the journal does not prescribe a specific tense for describing methods and datasets.

3.10) Line 244-246 “Next, we average the site-averaged TWL magnitudes within each region by regime, resulting in three regional TWL averages per region, one per regime.” ---- I see in line 251 you explain that you aren’t including inundation. I would move this to follow the pasted statement. As the reader I was confused for half the paragraph why you weren’t assessing four regimes.

Response: We revised this paragraph as suggested (lines 241 -245).

3.11) Line 256: should write your methods / analysis in past tense: “we evaluated”

Response: Please refer to response 3.9.

3.12) Line 260: “The percentiles indicate how frequently the TWL or dynamic SWL is to reach a threshold, allowing us to identify regions with higher potential for experiencing coastal impacts” replace “is to reach” with “reaches”

Response: We revised this sentence as suggested (lines 257 - 259).

3.13) Line 268: “Overall, the median and standard deviation of TWL, SWL, and wave runup magnitudes gradually increase from swash to overtopping.” Are these values reported somewhere?

Response: While the distributions for TWL, SWL, and wave runup are displayed in Figure 3, the values associated with the median and standard deviations were not previously reported in the first version of this manuscript. We have now added a table to the supporting information material (Table S5), and we refer to this table in the main text for the referred sentence (lines 264 - 266).

3.14) Figure 4: please make the legend (and it’s text) larger.

Response: We have updated the font size on Figure 4.

3.15) Line 380: “…SWL relative contribution begins to offset that of wave runup…”  Please be explicit about what regimes (or time periods?) you are comparing. Maybe could phrase as “Like the swash regime, TWLs in the collision regime are primarily driven by waves” (If I am interpreting this sentence correctly)

Response: We revised this sentence, which now is split into two, and added a reference to Figures 3 and 4 to improve sentence clarity (lines 367 - 370): *“TWLs in the collision regime remain primarily wave-driven (Figure 4), as waves become more energetic during storms (e.g., Bromirski & Kossin, 2008; Marsooli & Lin, 2018). However, waves relative contribution is generally offset by increasing SWL magnitude and contribution as TWLs shift into higher-impact regimes (Figures 3 and 4).”*

**References**

1. Ahn, S., Haas, K.A., Neary, V.S., 2020. Wave energy resource characterization and assessment for coastal waters of the United States. Appl. Energy 267, 114922. https://doi.org/10.1016/j.apenergy.2020.114922
2. Almar, R., Ranasinghe, R., Bergsma, E.W.J., Diaz, H., Melet, A., Papa, F., Vousdoukas, M., Athanasiou, P., Dada, O., Almeida, L.P., Kestenare, E., 2021. A global analysis of extreme coastal water levels with implications for potential coastal overtopping. Nat. Commun. 2021 121 12, 1–9. https://doi.org/10.1038/s41467-021-24008-9
3. Cohn, N., Ruggiero, P., García-Medina, G., Anderson, D., Serafin, K.A., Biel, R., 2019. Environmental and morphologic controls on wave-induced dune response. Geomorphology 329, 108–128. https://doi.org/10.1016/J.GEOMORPH.2018.12.023
4. Gallien, T.W., 2016. Validated coastal flood modeling at Imperial Beach, California: Comparing total water level, empirical and numerical overtopping methodologies. Coast. Eng. 111, 95–104. https://doi.org/10.1016/j.coastaleng.2016.01.014
5. Gomes da Silva, P., Coco, G., Garnier, R., Klein, A.H.F., 2020. On the prediction of runup, setup and swash on beaches. Earth-Sci. Rev. 204, 103148. https://doi.org/10.1016/j.earscirev.2020.103148
6. Guza, R.T., Feddersen, F., 2012. Effect of wave frequency and directional spread on shoreline runup. Geophys. Res. Lett. 39. https://doi.org/10.1029/2012GL051959
7. Hames, D.P., Vidal, I., Gouldby, B.P., 2023. Impacts of sea level rise on wave overtopping rates around the coast of England. Proc. Inst. Civ. Eng. - Marit. Eng. 176, 110–117. https://doi.org/10.1680/jmaen.2021.020
8. Hsu, C.-E., Serafin, K.A., Yu, X., Hegermiller, C.A., Warner, J.C., Olabarrieta, M., 2023. Total water levels along the South Atlantic Bight during three along-shelf propagating tropical cyclones: relative contributions of storm surge and wave runup. Nat. Hazards Earth Syst. Sci. 23, 3895–3912. https://doi.org/10.5194/nhess-23-3895-2023
9. Itzkin, M., Palmsten, M.L., Buckley, M.L., Birchler, J.J., Torres-Garcia, L.M., 2025. Developing a decision tree model to forecast runup and assess uncertainty in empirical formulations. Coast. Eng. 195, 104641. https://doi.org/10.1016/j.coastaleng.2024.104641
10. Kim, L.N., Brodie, K.L., Cohn, N.T., Giddings, S.N., Merrifield, M., 2023. Observations of beach change and runup, and the performance of empirical runup parameterizations during large storm events. Coast. Eng. 184, 104357. https://doi.org/10.1016/j.coastaleng.2023.104357
11. Leaman, C.K., 2020. py-wave-runup: Empirical wave runup models implemented in Python.
12. Lehmann, M., Karimpour, F., Goudey, C.A., Jacobson, P.T., Alam, M.-R., 2017. Ocean wave energy in the United States: Current status and future perspectives. Renew. Sustain. Energy Rev. 74, 1300–1313. https://doi.org/10.1016/j.rser.2016.11.101
13. Leung, M., Cagigal, L., Mendez, F., Ruggiero, P., 2024. Projecting Future Chronic Coastal Hazard Impacts, Hotspots, and Uncertainty at Regional Scale. Earths Future 12, e2024EF005523. https://doi.org/10.1029/2024EF005523
14. Matias, A., Williams, J.J., Masselink, G., Ferreira, Ó., 2012. Overwash threshold for gravel barriers. Coast. Eng., BARDEX: a large-scale laboratory study of gravel barrier dynamics 63, 48–61. https://doi.org/10.1016/j.coastaleng.2011.12.006
15. NOAA CO-OPS, 2025. CO-OPS Products - NOAA Tides & Currents [WWW Document]. URL https://tidesandcurrents.noaa.gov/products.html (accessed 4.12.23).
16. Oliver, T.S.N., Kinsela, M.A., Doyle, T.B., McLean, R.F., 2024. Foredune erosion, overtopping and destruction in 2022 at Bengello Beach, southeastern Australia. Camb. Prisms Coast. Futur. 2, e7. https://doi.org/10.1017/cft.2024.8
17. Palmsten, M.L., Holman, R.A., 2012. Laboratory investigation of dune erosion using stereo video. Coast. Eng. 60, 123–135. https://doi.org/10.1016/j.coastaleng.2011.09.003
18. Park, H., Cox, D.T., 2016. Empirical wave run-up formula for wave, storm surge and berm width. Coast. Eng., Swash-zone Processes 115, 67–78. https://doi.org/10.1016/j.coastaleng.2015.10.006
19. Parker, K., Erikson, L., Thomas, J., Nederhoff, K., Barnard, P., Muis, S., 2023. Relative contributions of water-level components to extreme water levels along the US Southeast Atlantic Coast from a regional-scale water-level hindcast. Nat. Hazards 2023 1–30. https://doi.org/10.1007/S11069-023-05939-6
20. Plant, N.G., Stockdon, H.F., 2012. Probabilistic prediction of barrier-island response to hurricanes. J. Geophys. Res. Earth Surf. 117. https://doi.org/10.1029/2011JF002326
21. Quadrado, G.P., Serafin, K.A., 2024. The Timing, Magnitude, and Relative Composition of Extreme Total Water Levels Vary Seasonally Along the U.S. Atlantic Coast. J. Geophys. Res. Oceans 129, e2023JC020557. https://doi.org/10.1029/2023JC020557
22. Sallenger, A.H., 2000. Storm Impact Scale for Barrier Islands on JSTOR. J. Coast. Res. 16, 890–895.
23. Serafin, K.A., Ruggiero, P., Stockdon, H.F., 2017. The relative contribution of waves, tides, and nontidal residuals to extreme total water levels on U.S. West Coast sandy beaches. Geophys. Res. Lett. 44, 1839–1847. https://doi.org/10.1002/2016GL071020
24. Stockdon, H.F., Doran, K.S., Thompson, D.M., Sopkin, K., L., Plant, N.G., Sallenger, A.H., 2012. National Assessment of Hurricane-Induced Coastal Erosion Hazards: Gulf of Mexico (Open-File Report), Open-File Report. USGS.
25. Stockdon, H.F., Sallenger, A.H., Holman, R.A., Howd, P.A., 2007. A simple model for the spatially-variable coastal response to hurricanes. Mar. Geol. 238, 1–20. https://doi.org/10.1016/j.margeo.2006.11.004
26. Stockdon, H.F., Thompson, D.M., Plant, N.G., Long, J.W., 2014. Evaluation of wave runup predictions from numerical and parametric models. Coast. Eng. 92, 1–11. https://doi.org/10.1016/j.coastaleng.2014.06.004
27. Talke, S.A., 2025. How tidal properties influence the future duration of coastal flooding. Npj Nat. Hazards 2, 36. https://doi.org/10.1038/s44304-025-00086-3
